# Bi‐Functional Materials for Sulfur Cathode and Lithium Metal Anode of Lithium–Sulfur Batteries: Status and Challenges

**DOI:** 10.1002/advs.202407304

**Published:** 2024-10-16

**Authors:** Ying Dou, Junling Guo, Junke Shao, Jiaozi Duan, Huan Liang, Xing Cheng, Yanbing He, Jinping Liu

**Affiliations:** ^1^ Country State Center for International Cooperation on Designer Low carbon & Environmental Materials School of Materials Science and Engineering Zhengzhou University 100 Kexue Avenue Zhengzhou 450001 P. R. China; ^2^ Shenzhen All‐Solid‐State Lithium Battery Electrolyte Engineering Research Center Institute of Materials Research (IMR) Tsinghua Shenzhen International Graduate School Tsinghua University Shenzhen 518055 P. R. China; ^3^ School of Chemistry Chemical Engineering and Life Science State Key Laboratory of Advanced Technology for Materials Synthesis and Processing Wuhan University of Technology Wuhan 430070 P. R. China

**Keywords:** bi‐functional materials, high energy density, lithium dendrites, lithium–sulfur batteries, shuttle effects

## Abstract

Over the past decade, the most fundamental challenges faced by the development of lithium–sulfur batteries (LSBs) and their effective solutions have been extensively studied. To further transfer LSBs from the research phase into the industrial phase, strategies to improve the performance of LSBs under practical conditions are comprehensively investigated. These strategies can simultaneously optimize the sulfur cathode and Li‐metal anode to account for their interactions under practical conditions, without involving complex preparation or costly processes. Therefore, “two‐in‐one” strategies, which meet the above requirements because they can simultaneously improve the performance of both electrodes, are widely investigated. However, their development faces several challenges, such as confused design ideas for bi‐functional sites and simplex evaluation methods (i. e. evaluating strategies based on their bi‐functionality only). To date, as few reviews have focused on these challenges, the modification direction of these strategies is indistinct, hindering further developments in the field. In this review, the advances achieved in “two‐in‐one” strategies and categorizing them based on their design ideas are summarized. These strategies are then comprehensively evaluated in terms of bi‐functionality, large‐scale preparation, impact on energy density, and economy. Finally, the challenges still faced by these strategies and some research prospects are discussed.

## Graphical abstract

Emergent advancements in “two‐in‐one” strategies for LSBs are reviewed and classified based on their design ideas. Then, the strengths and weaknesses of these strategies are comprehensively evaluated in terms of bifunctionality, large‐scale preparation, impact on battery energy density, and economy.

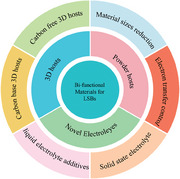

## 1 Introduction

**Figure 1:**
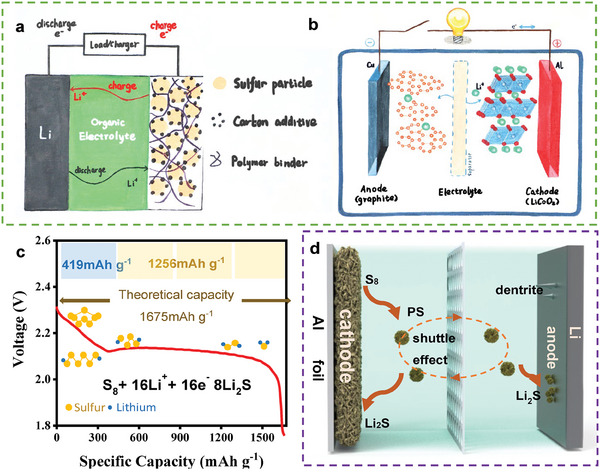
a) Freehand diagram of the operating principle of a) LSBs and b) LIB. c) Typical discharge curve of LSBs. d) Schematic graph of the reaction process of LSBs. a) Reproduced with permission^[^ ^4^ ^]^ Copyright 2014, American Chemical Society. b) Reproduced with permission^[^ ^5^ ^]^ Copyright 2013, American Chemical Society.

With the development of society, energy storage devices with high energy density are urgently required to meet the growing demand for emerging applications. Lithium–sulfur batteries (LSBs) have attracted attention as one of the most promising next‐generation batteries owing to their high theoretical energy density (2600 Wh kg^−1^),^[^ ^1^, ^2^, ^3^ ^]^ which is attributed to their unique operating reaction (**Figure** **1**a)^[^ ^4^ ^]^ that is quite different from the intercalation–deintercalation electrochemical reaction of lithium‐ion batteries (Figure 1b).^[^ ^5^ ^]^ The electrode reaction of LSBs is based on the direct reaction between sulfur (theoretical specific capacity = 1675 mAh g^−1^) and metallic lithium (Li, theoretical specific capacity = 3860 mAh g^−1^).^[^ ^6^ ^]^ Typically, there are two clear plateaus in the discharge curve of LSBs (Figure 1c). These plateaus correspond to the two reduction stages of the electrode reaction of LSBs (S_8_+16Li^+^+16e^−^→8Li_2_S). The first plateau (at 2.1–2.4 V) can be attributed to the formation and conversion of soluble high‐order Li polysulfides (PSs, S_8_+2Li^+^+2e^−^→Li_2_S_8_; S_8_^2−^→S_6_^2−^→ S_4_^2−^). The second one (at ≈2.1 V) can be attributed to the formation of insoluble low‐order Li compounds (Li_2_S_4_+2Li^+^+2e^−^→2Li_2_S_2_; Li_2_S_2_+2Li^+^+2e^−^→2Li_2_S).^[^ ^7^ ^]^

However, this unique electrode reaction has numerous and serious drawbacks that restrict the development of LSBs. Some of these drawbacks are summarized below.^[^ ^8^, ^9^ ^]^ 1) LSBs exhibit low sulfur utilization owing to the intrinsic insulation of S_8_ and its insoluble products.^[^ ^10^ ^]^ The second reduction stage, which contributes up to 3/4 (1200 mAh g^−1^) of the theoretical capacity of LSBs, involves a solid reaction.^[^ ^11^ ^]^ Therefore, the intrinsic insulativity of S_8_ and its insoluble products severely affects the reaction kinetics and reduces the sulfur utilization rate. 2) The cathode structure is fragile because of the large volume change (≈79%) during the charge–discharge process, which results from the density difference between S_8_ (2.07 g cm^−3^) and Li_2_S (1.66 g cm^−3^).^[^ ^12^ ^]^ 3) LSBs exhibit rapid capacity decay owing to the shuttle effect of PSs. The appearance of soluble PSs prevents the deposition of insulating products on the conductive material, thereby ensuring the subsequent reaction of sulfur. However, soluble PSs can “shuttle” between the sulfur cathode and Li‐metal anode under the effect of concentration difference (Figure 1d).^[^ ^13^ ^]^ During this “shuttle” process, the PSs that reach the Li‐metal anode under the shuttle effect can react with metallic Li to form insoluble low‐order sulfides (Li_2_S_2_/Li_2_S), leading to an irreversible sulfur loss.^[^ ^14^, ^15^, ^16^ ^]^ 4) The series of problems existing in the Li‐metal anode also seriously inhibit the development of LSBs. Li‐metal anode is difficult to be replaced in LSBs. In the electrode reaction of LSBs, sulfur needs to get Li ions at first, featuring a typical anode reaction. The anode materials commonly used in lithium‐ion batteries (also featuring anode reaction) do not match the sulfur cathodes. Therefore, the issues of the Li metal anode also greatly affect the performance of LSBs^17^. Typically, the natural solid electrolyte interface (SEI) formed by the spontaneous side reactions of the electrolyte with Li metal is often multicomponent and inhomogeneous, resulting in differences in ion transport and mechanical properties spatially. In LSBs, due to the “shuttle effect”, the inner layer of SEI is enriched in Li_2_S_2_/Li_2_S, thereby exacerbating the above differences. Such differences easily lead to the formation and growth of Li dendrites, which can cause the following issues. First, the natural SEI can be broken by Li dendrites, resulting in continuous side reactions.^[^ ^18^ ^]^ Second, Li dendrites are prone to fracture during repeated deposition/stripping processes. These fractured Li can be completely coated by SEI and then form “dead Li” that cannot be reused, causing low Li utilization. Third, the inhomogeneous deposition of Li easily leads to the production of porous Li and thick SEI, giving rise to increased polarization. Finally, overgrown Li dendrites may puncture the separator and then short‐circuit the cell, leading to serious security concerns.^[^ ^19^ ^]^

The following are common strategies developed in the past decade to address these problems: 1) Novel hosts are designed and introduced into cathodes or separators. These hosts can provide an ideal place for sulfur reactions owing to their high electrical conductivity or numerous catalytic sites, accommodate the volume change of sulfur, and trap PSs through physical and chemical adsorption or confinement effect.^[^ ^20^, ^21^, ^22^ ^]^ 2) A functional framework is developed to provide dendrite‐free Li‐metal anodes that can reduce local current density and promote Li nucleation.^[^ ^23^, ^24^ ^]^ 3) Functional electrolytes that can form dense passivation layers with high mechanical properties are introduced on the surface of cathodes or anodes to suppress the shuttle effect and growth of dendrites.^[^ ^25^ ^]^

**Figure 2:**
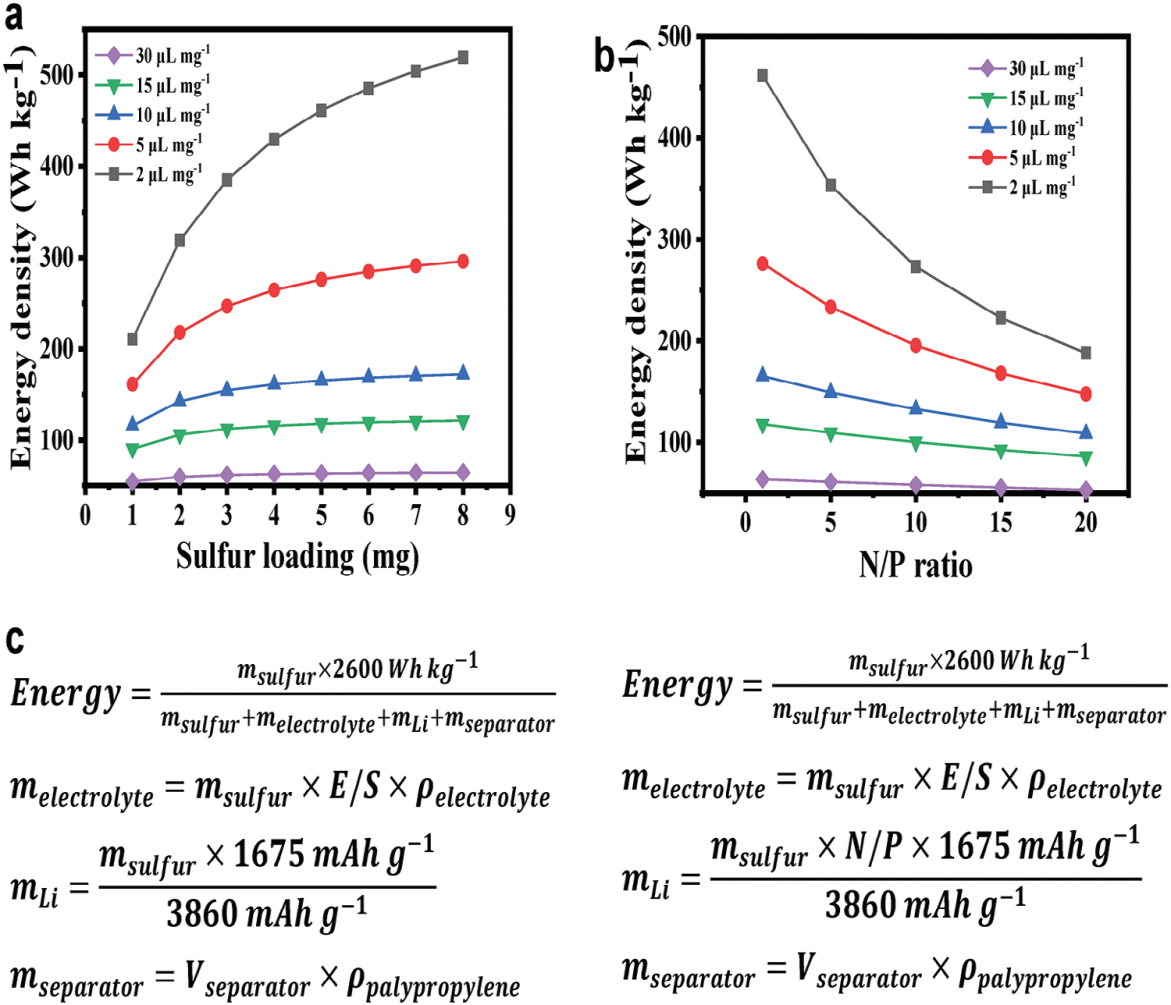
Energy density of LSBs with a) different sulfur loading and b) N/P ratio at various E/S ratios. c) Corresponding theoretical formulas and parameters. In the formulas, ρ_electrolyte_ is 1.261 g cm^−3^, *V*_separator_ is 20 µm × π × (9.5 mm)^[^ ^2^ ^]^ × 50% (porosity), and ρ_palypropylene_ is 0.93 g cm^−3^.

However, few strategies have been introduced to simultaneously enhance sulfur cathodes and Li‐metal anodes, although both affect the electrochemical performance of LSBs. This is because most of the above‐discussed strategies have been developed under testing conditions that stay away from practical application, such as high electrolyte‐to‐sulfur (E/S) ratios and large‐capacity ratios of negative‐to‐positive electrodes (N/P).^[^ ^26^, ^27^, ^28^ ^]^ Under these testing conditions, the interactions between the sulfur cathode and Li‐metal anode are weak; thus, these strategies rarely focus on improving the performance of both electrodes simultaneously. However, with the deepening of the research, researchers gradually realized that the energy density, which is the primary advantage of LSBs, can be seriously affected by the E/S (**Figure** **2**a) and N/P ratios (Figure 2b). The corresponding theoretical formulas and parameters are provided in Figure 2c. Therefore, to transfer LSBs from the laboratory to the industry, strategies to improve the performance of LSBs under practical conditions (E/S ratio ≤ 5 µL mg^−1^; N/P ratio ≤ 5) should be developed.^[^ ^29^, ^30^ ^]^

Under practical conditions, sulfur cathodes and Li‐metal anodes interact with each other. 1) At a low E/S ratio, the performance of sulfur cathodes is largely affected by unmodified Li‐metal anodes. The E/S ratio considerably affects the performance of the sulfur electrode. Insufficient electrolytes cannot completely dissolve insulating PSs, causing their deposition on the reaction surface, which results in an incomplete sulfur reaction.^[^ ^31^ ^]^ Moreover, using an insufficient electrolyte increases the concentration of PSs, which not only deteriorates the performances of electrolytes due to the increasing viscosity and decreasing ion conductivity but also intensifies the “shuttle effect” due to the increased concentration difference.^[^ ^32^ ^]^ Unmodified Li‐metal anodes lead to continuous consumption of the available electrolyte (i.e., a continuously decreasing E/S ratio), as the natural SEI can be broken by Li dendrites. Thus, using unmodified Li‐metal anodes largely affects the performance of sulfur cathodes under a low E/S ratio.^[^ ^24^ ^]^ 2) At a low N/P ratio, unmodified Li‐metal anodes can greatly affect the sulfur utilization of the cathode. Due to the continuous irreversible side reaction between Li‐metal and the electrolyte, unmodified Li‐metal anodes exhibit low CE, leading to a continuous decline in the N/P ratio. When the N/P ratio is less than 1, it will greatly affect sulfur utilization.^[^ ^3^ ^]^ Moreover, during the above‐mentioned side reaction, an SEI that is continuously thickening and porous Li are generated, thus increasing polarization and affecting the sufficient reaction of sulfur. 3) At a low N/P ratio, an unmodified sulfur cathode can deteriorate the performance of Li‐metal anodes. The serious shuttle effect generated by the unmodified sulfur cathode results in a reaction between PSs and Li‐metal, and the formation of an uneven Li_2_S layer with a low Li^+^ diffusion coefficient (<10^−9^ S cm^−1^),^[^ ^33^ ^]^ resulting in a low CE of LSBs and uneven Li deposition on the well‐designed Li‐metal anodes. Therefore, to improve the performance of LSBs under practical conditions, it is necessary to modify the sulfur cathodes and Li‐metal anode simultaneously.

To avoid complex preparation processes and additional considerations to ensure compatibility between the improvement strategies of cathodes and anodes, it is essential to develop effective “two‐in‐one” strategies that can simultaneously optimize both electrodes for practical LSBs via using simple processing technologies. Some structures and materials have been widely used for the improvement of both the cathode and anode. However, they cannot be directly used for “two‐in‐one” strategies owing to some drawbacks, which are summarized as follows:

1. Three‐dimensional (3D)‐structured hosts can accommodate the volume expansion of sulfur and reduce the local current density. Thus, they are widely used in both cathodes and anodes.^[^ ^34^ ^]^ However, materials that can easily form 3D structures do not typically exhibit bi‐functionality, i.e., simultaneously suppressing the shuttle effect and formation of Li dendrites. Thus, they cannot be used alone to effectively improve the performance of LSBs under practical conditions.
2. Using various single‐functional materials (for cathode or anode) in one host can simultaneously prevent the shuttle effect and dendrite formation without complex design. However, it is difficult to achieve a synergetic effect (i.e., “one plus one is greater than two”) among different types of functional materials. Therefore, large amounts of these materials should be used to ensure that they fulfill both functions, leading to higher host masses, which are likely to limit the energy density of LSBs.
3. Bi‐functional materials, such as some types of doped carbons and heterojunctions, have been reported to simultaneously suppress the shuttle effect (via trapping PSs or accelerating the conversion of PSs to Li_2_S) and inhibit the formation of Li dendrites (through their lithiophilic sites, which contribute to uniform Li deposition).^[^ ^35^, ^36^, ^37^, ^38^ ^]^ However, as yet, there are no reports of bi‐functional materials that can be prepared at scale. Furthermore, there is no clear interpretation of the mechanism of their dual effect, making it difficult to select and modify these materials.
4. Stable and compact artificial interfaces with superior mechanical properties can be formed on both electrodes by optimizing the electrolytes. These are often used to improve the performance of LSBs under practical conditions.^[^ ^39^ ^]^ Owing to the physical confinement effect of artificial SEIs, they can suppress the shuttle effect because of their compactness and inhibit the growth of Li dendrites because of their superior mechanical properties. However, the manufacturing process of these interfaces cannot be controlled, limiting their application in the simultaneous improvement of both electrodes.

Nowadays, the necessity of developing “two‐in‐one” strategies is now being recognized by researchers, and considerable attention have been devoted to modifying these strategies and developing novel strategies. However, some challenges, such as confused design ideas for bi‐functional sites and simplex evaluation methods (i. e. evaluating strategies based on their bi‐functionality only), face the development of these strategies. To date, as few reviews have focused on these challenges, the modification direction of these strategies is indistinct, hindering further developments in the field. In this review, we summarize the advances achieved in the “two‐in‐one” strategies and divide them into four categories based on their design concept: 1) optimization of the 3D hosts by introducing bi‐functional sites; 2) design of high‐efficiency bi‐functional powder materials through size effects or by regulation of electron distribution; and 3) developing bi‐functional electrolytes via film‐forming additives or optimizing ions transport. 4) Modifying the separators using two single‐functional materials without any synergistic effect. This method avoids complex designs and is compatible with existing industrial equipment. We comprehensively evaluated the strengths and weaknesses of these methods based on their bi‐functionality, compatibility with large‐scale manufacturing, impact on energy density, and economy. Finally, the challenges and some of the research prospects are discussed.

## 2 Optimization of 3D Hosts

3D‐structured hosts were selected for the design of “two‐in‐one” strategies because they can accommodate the volume expansion of sulfur/Li and reduce the local current density in LSBs. Moreover, they can be easily manufactured. However, they generally do not exhibit bi‐functionality; therefore, several modification methods have been developed to solve this problem.

### 2.1 3D Doped Carbon Hosts

Carbon materials obtained through polymerization carbonization can be used to prepare various highly conductive 3D hosts such as ordered 3D macroporous structures, self‐supporting fibers, and 3D‐printed self‐supporting structures.^[^ ^40^, ^41^, ^42^, ^43^ ^]^ However, their bi‐functionality, i.e., their catalytic activity for PSs and affinity for Li^+^, is generally poor^[^ ^44^ ^]^ owing to their weak polarity, which considerably affects their ability to inhibit the shuttle effect and formation of Li dendrites.

**Figure 3:**
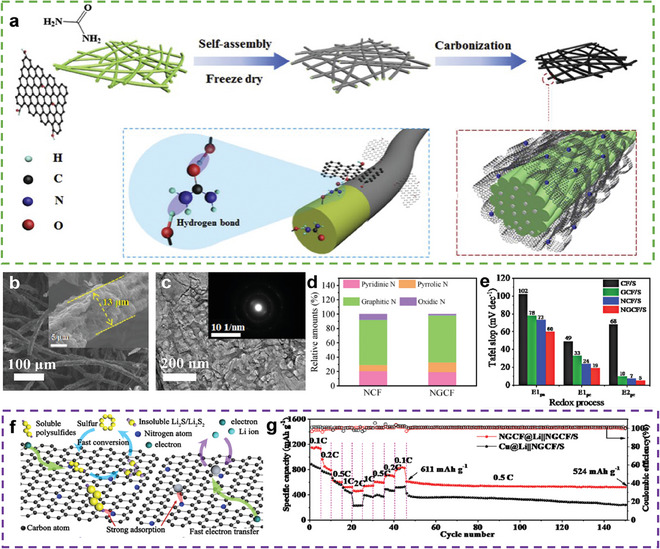
a) Schematic illustration of the fabrication process of NGCF. b) SEM images of NGCF. c) TEM image of NGCF, (inset: SAED pattern). d) Comparison of nitrogen species relative amounts in NGCF and GCF. e) Comparison of Tafel slop of various redox processes on different samples. f) Illustration of the PSs immobilization and conversion, the uniform of Li nuclei, and Li deposition on the surface of NGCF. g) Rate and cycling performances of the NGCF@Li || NGCF/S and Cu@Li||NGCF/S full cells. (a–g) Reproduced with permission^[^ ^45^ ^]^ Copyright 2020, Elsevier.

Introducing heteroatoms into carbon materials can alter the electronic structure of carbon. This is an effective approach to modifying the bi‐functionality of carbon materials. Wang et al. prepared a 3D cable‐like material composed of porous fibrous carbon and N‐doped graphene foam (NGCF) (**Figure** **3**a–c) as hosts for both the cathode and anode.^[^ ^45^ ^]^ Owing to the higher ratio of pyrrolic N in NGCF (Figure 3d), it exhibited catalytic activity for the sulfur reaction (Figure 3e) and a strong affinity for Li^+^. In addition, the large surface area and numerous pores offer adequate space for volume expansion and reaction sites for sulfur and Li (Figure 3f). Therefore, using this host improved the rate performance of the NGCF@Li || NGCF/S cells (462 mAh g^−1^ at 2 C) along with a stable cycle performance (capacity retention after 100 cycles at 0.5 C = 85.76%) (Figure 3g). However, even at a high‐sulfur loading (6.8 mg cm^−2^) and an E/S ratio of 20 µL mg^−1^, the areal capacity of the NGCF@Li || NGCF/S cells was still less than 5 mAh cm^−2^, indicating that the bi‐functionality of this heteroatom‐doped carbon cannot meet practical application requirements.

The low bi‐functionality of doped carbon may be attributed to the limited optimization of their electronic structures. Two methods are typically used to address this problem. One method is to design carbon with numerous doping sites. However, this is achieved under harsh preparation conditions and can easily affect the conductivity of the material.^[^ ^46^ ^]^ The other method involves designing carbon with edge‐doping sites,^[^ ^47^ ^]^ leading to additional uncoordinated electron pairs. Edge‐doping sites are typically introduced into carbon materials by increasing their specific surface areas or introducing numerous micropores into the material. However, these methods result in incomplete wetting of the hosts by the electrolyte, considerably limiting the sulfur and Li utilizations because Li ions cannot be transported to unwetted surfaces to participate in electrode reactions. Therefore, this modification method requires further investigation under practical conditions.

### 2.2 3D Carbon Hosts Loading Functional Materials

The low bi‐functionality of doped carbon is attributed to its nonpolar electronic structure of carbon, which is difficult to completely overcome by simple doping. Thus, various functional materials that can provide better active sites for the reaction of sulfur and Li metal are introduced into 3D‐carbon hosts. These active sites are generated by the functional materials or as a result of their interaction with nearby carbon atoms.

#### 2.2.1 Synergetic Effect of Two Single‐functional Materials

Based on the synergetic effect of single‐functional materials (i.e., “one plus one greater than two”), some significant bi‐functional hosts have been developed.

**Figure 4:**
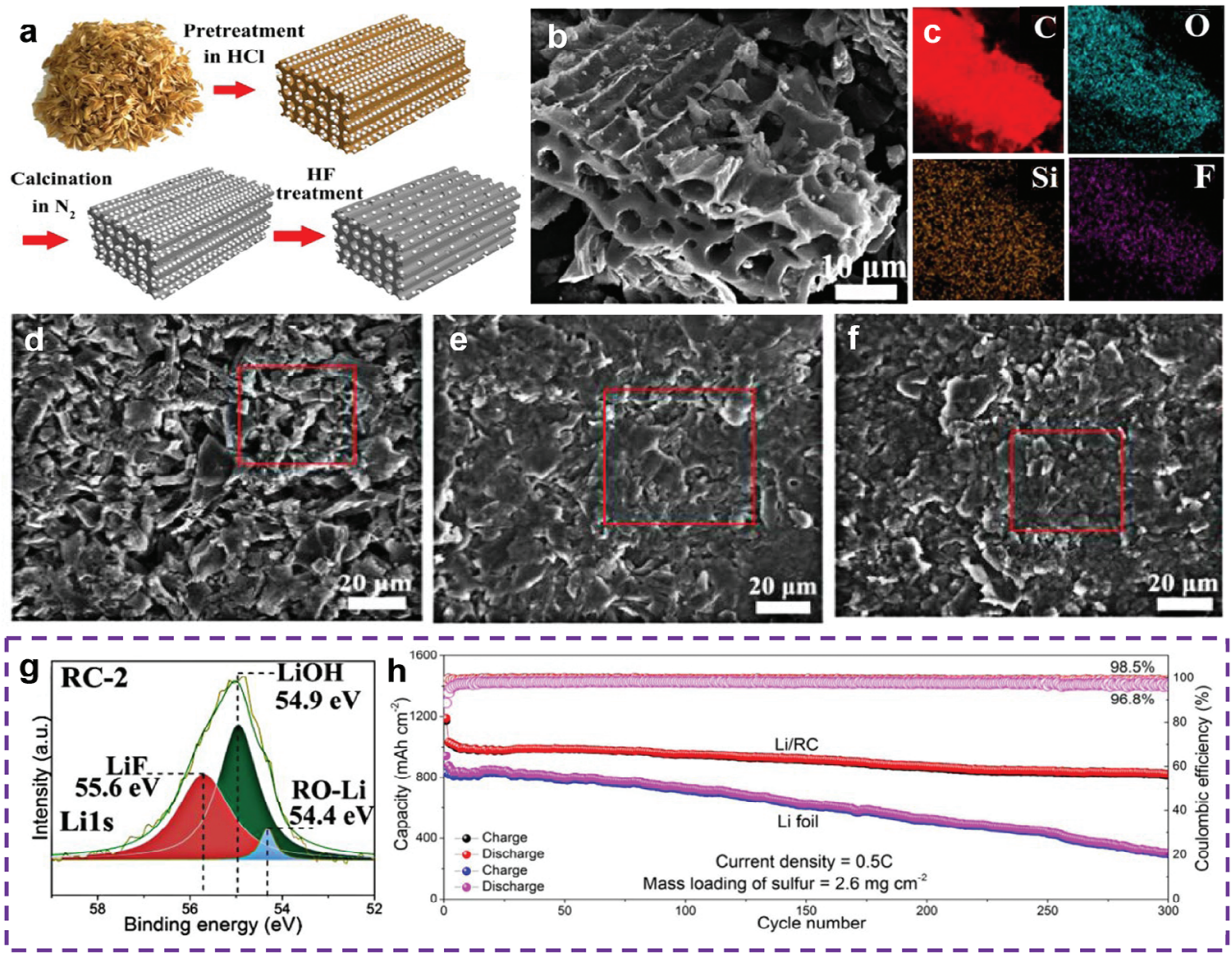
a) Schematic illustration of the synthesis procedure of RC samples. b,c) SEM images and elemental mapping of RC‐2 sample. Top‐view of RC‐2 d) at the beginning, e) after plating 1 mA h cm^−2^ Li, and f) after stripping 1 mA h cm^−2^ Li. g) XPS characterization of the SEI layer. h) Cycle performance of Li/RC||S/RC and Li||S/RC cells. (a–h) Reproduced with permission^[^ ^48^ ^]^ Copyright 2018, Elsevier.

Tao et al. reported rice husk–derived carbon (RC) as a bi‐functional host for LSBs^48^. The steps for the synthesis of RC are shown in **Figure** **4**a. The RC treated with HF exhibited a porous channel structure with no SiO_2_ nanoparticles on its surface (Figure 4b). Moreover, the RC surface was modified by the F‐containing groups (Figure 4c). As shown in Figure 4d–f, the surface morphology of the Li‐metal anode based on RC (Li/RC) before and after cycling (capacity = 1 mAh cm^−2^) hardly changed, indicating a uniform Li deposition on the RC host. This is because SiO_2_ nanoparticles serve as lithiophilic nucleation sites to control the deposition of Li, and the F‐containing groups participate in the formation of a rough F‐rich SEI (Figure 4g), which can suppress the formation of Li dendrites. In addition, as a sulfur host, the porous channels of RC provide abundant space to accommodate the volume change of sulfur and numerous high‐conductivity reaction sites. The trapping of PSs occurs via polar–polar interactions between them and the SiO_2_ nanoparticles inside the pores as well as the F‐containing groups on the surface of the modified RC. Thus, the capacity of Li/RC||S/RC cells with a sulfur loading of 2.6 mg cm^−2^ and an E/S ratio of 19.2 µL mg^−1^ remains 800 mAh g^−1^ after 300 cycles at 0.5 C (Figure 4h).

**Figure 5:**
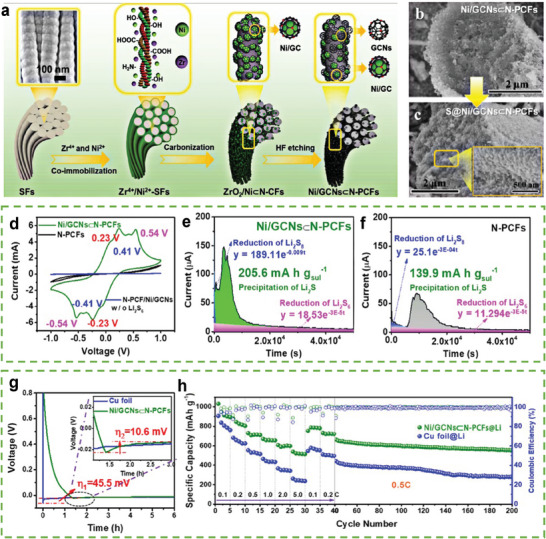
a) Schematic depiction of the preparation of Ni/GCNs⊂N‐PCFs. Cross–sectional FESEM images of b) Ni/GCNs⊂N‐PCFs and c) S@Ni/GCNs⊂N‐PCFs. d) CV curves of symmetrical cells with Ni/GCNs⊂N‐PCF and N‐PCF electrodes in the electrolyte with or without Li_2_S_6_. Chronoamperometry curves of a Li_2_S_8_/tetraglyme solution at 2.05 V on the surfaces of e) Ni/GCNs⊂NPCF and f) N‐PCF cathodes. g) Voltage profiles of Li depositing on Ni/GCNs⊂N‐PCFs and Cu foil electrodes at 0.5 mA cm^−2^. h) Electrochemical performance of LSBs using S@Ni/GCNs⊂N‐PCF cathodes and Ni/GCNs⊂N‐PCFs@Li. (a–h) Reproduced with permission^[^ ^49^ ^]^ Copyright 2020, American Chemical Society.

In addition to the synergies between different functions in a single material, synergies can be directly formed between materials. Zhang confirmed this synergetic effect through the in situ preparation of a skin fiber–derived super hierarchical N‐doped porous carbon framework (N‐PCF) implanted with nickel and graphitic carbon nanocages (Ni/GCNs⊂N‐PCFs, **Figure** **5**a–c).^[^ ^49^ ^]^ The electropositive Ni nanoparticles can enhance the adsorption of PSs and accelerate their redox kinetics through electron transfer from Ni nanoparticles to the N‐doped graphene, and the Li affinity can be enhanced by the electrons obtained from these lithiophilic N sites. The catalytic activity of the PS conversion was confirmed using cyclic voltammetry (CV) (Figure 5d), which indicates that the response current of Ni/GCNs⊂N‐PCFs is the largest. The potentiostatic discharge curve of Ni/GCNs⊂N‐PCFs (Figure 5e) exhibited an earlier deposition peak and a considerably larger area than those of N‐PCF (Figure 5f), indicating the facile nucleation kinetics of Li_2_S on Ni/GCNs⊂N‐PCFs. Owing to the lithiophilic N sites around Ni, the overpotential of the Li nucleation on Ni/GCNs⊂N‐PCFs is lower than that on Cu foil (Figure 5g), resulting in a smoother Li deposition on Ni/GCNs⊂N‐PCFs. Thus, full cells based on Ni/GCNs⊂N‐PCF hosts (sulfur loading = 1.5 mg cm^−2^ and E/S ratio = 30 µL mg^−1^) exhibit superior rate performance for both electrodes (555 mAh g^−1^ at 5 C) and high cycling stability (99.4% capacity retention after 160 cycles at 0.5 C) (Figure 5h).

#### 2.2.2 Bi‐functional Materials

Using one material to improve both the cathode and anode of LSBs can reduce the mass of inactive materials (which does not provide capacity in LSBs) compared to that required using the above strategy. Therefore, research has been focusing on introducing various types of bi‐functional materials into 3D‐carbon hosts.

**Figure 6:**
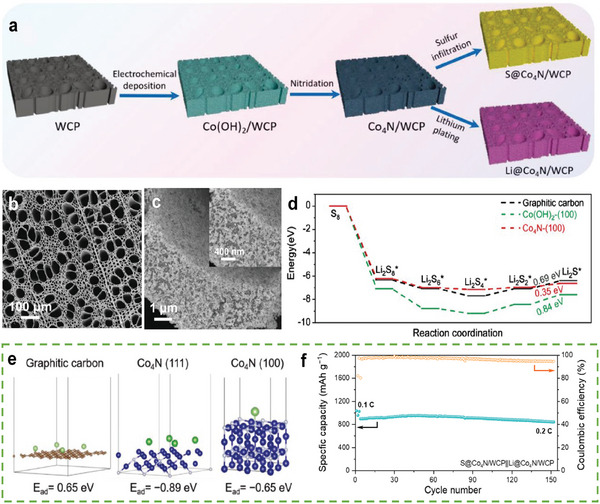
a) Schematic illustration of the fabrication of Co_4_N/WCP host. b,c) SEM images of Co_4_N/WCP. d) Calculated Gibbs free energies for the reduction of S_8_ on different substrates. e) Optimized adsorption structures and adsorption energies for Li on different surfaces of Co_4_N and WCP substrates. f) Cycle performance of LSBs using Co_4_N/WCP with a sulfur loading of 4 mg cm^−2^ at 0.2 C. (a–f) Reproduced with permission^[^ ^50^ ^]^ Copyright 2022, Wiley‐VCH.

Liu et al. reported a host based on a vertically aligned Co_4_N nanoparticle–decorated 3D carbon plate derived from wood (Co_4_N/WCP) that can improve Li deposition and enhance cathodic electrocatalysis.^[^ ^50^ ^]^ The synthesis steps for this host material are shown in **Figure** **6**a. The interconnected 3D structure of the host, which contains meso‐ and macro‐ channels, can optimize the electron transmission and electrolyte distribution in LSBs (Figure 6b,c). As shown in Figure 6d, the energy barrier of the rate‐limiting step for the sulfur reaction on Co_4_N is lower than that on other surfaces. This is because Co–N bonding generates a synergistic effect on the Co_4_N surface, where unsaturated bonds can change the electronic structure and considerably increase the redox kinetics. Moreover, the binding energy of Li ions on Co_4_N was higher (Figure 6e) owing to the lithiophilic nature of Co_4_N nanoparticles, which may be attributed to Co. Thus, the Co_4_N nanoparticles in the 3D structure can provide catalytic sites for sulfur and deposition sites for Li. Therefore, Co_4_N/WCP is an efficient bi‐functional host that enables LSBs with a sulfur loading of 4 mg cm^−2^ to retain a high capacity after 150 cycles at 0.2 C (842.9 mAh g^−1^, capacity decay of 0.043% per cycle, Figure 6f).

**Figure 7:**
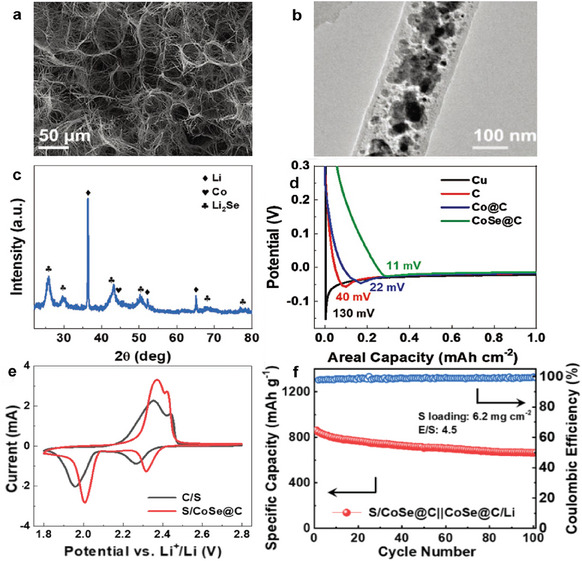
a) SEM and b) TEM images of CoSe@C nanowires. c) XRD pattern of CoSe@C/Li. d) Voltage profiles during initial Li plating on different substrates at 1 mA cm^−2^. e) CV curves of the S/CoSe@C and C/S cathodes at 0.1 mV s^−1^. f) The areal capacity of the S/CoSe@C||CoSe@C/Li cell at 0.2C rate with a high sulfur loading of 6.2 mg cm^−2^ per cell. (a–f) Reproduced with permission^[^ ^51^ ^]^ Copyright 2020, Wiley‐VCH.

In addition, there is another class of materials, whose bi‐functions come from the synergy of themselves and their in situ products, which need attention. Manthiram et al. prepared a 3D framework with CoSe particles coupled with conductive carbon nanowires (**Figure** **7**a,b) as a “two‐in‐one” host for both electrodes.^[^ ^51^ ^]^ As shown in Figure 7c, during the initial Li nucleation, CoSe in the host for Li metal anode will be transformed into Co and Li_2_Se. The abundant Co formed in situ can act as Li‐nucleation sites, and Li_2_Se can enhance the ionic conductivity of the LSBs. Therefore, the Li‐nucleation overpotential on this host was much lower than that on other hosts (Figure 7d). In the cathode (Figure 7e), the CV of S/CoSe@C exhibited low polarization and high peak intensity, suggesting that CoSe can greatly accelerate the conversion kinetics of the sulfur reaction. Thus, even under practical conditions with a high‐sulfur loading (6.2 mg cm^−2^) and a low E/S ratio (4.5 µL mg^−1^), using this host results in high‐capacity LSBs (860 mAh g^−1^) with a high‐capacity retention of ≈70% after 100 cycles at 0.2 C (Figure 7f).

However, the practical application of these 3D carbon‐based bi‐functional hosts under practical conditions is still restricted. Carbon substrate remain weak interactions with polar PSs in the cathode^[^ ^52^ ^]^ and low affinity to Li in the anode.^[^ ^53^ ^]^ Thus, a high quantity of functional materials should be loaded into the 3D structure to guarantee the efficient bi‐functionality of these hosts. Meanwhile, the structure of 3D‐carbon hosts generally features low specific surface areas and macropores (which is required to ensure the complete wettability of hosts at low E/S ratios) resulting in a low confinement effect. Thus, adequate dosages of uniformly dispersed, small‐sized functional materials are difficult to load on these 3D‐carbon hosts. This is because these functional materials easily agglomerated during the high‐temperature annealing process, which is a necessary process to ensure the conductivity of 3D‐carbon hosts. Currently, preparing efficient bi‐functional hosts for low E/S ratios based on this strategy is still a challenge.

### 2.3 Carbon‐free 3D Hosts

**Figure 8:**
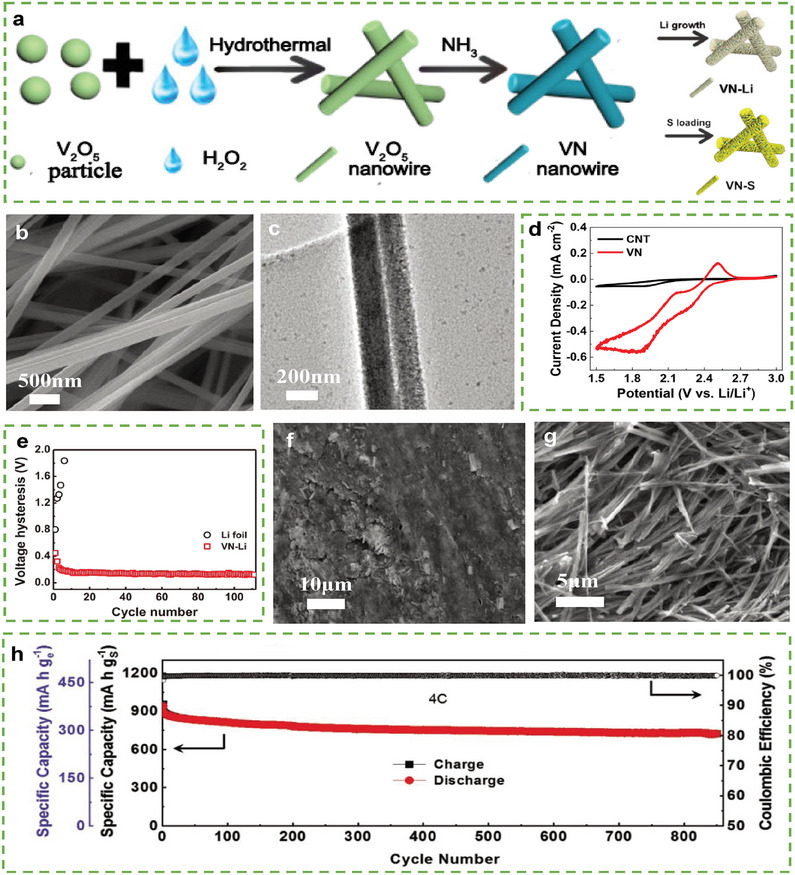
a) Schematic illustration of the fabrication procedure of VN‐Li and VN‐S. b,c) SEM and Low‐magnification TEM images of the VN aerogel. d) CV curves of CNT and VN with the catholyte solution in a three‐electrode system. e) Average voltage hysteresis of the symmetric cells with VN‐Li and Li foil at 10 mA cm^−2^/10 mA h cm^−2^. SEM images of VN after Li plating f) and Li stripping g) process with a capacity of 10 mA h cm^−2^. h) Long‐term cycling performance of the VN‐Li||VN‐S cell at 4 C with 850 cycles. (a–h) Reproduced with permission^[^ ^54^ ^]^ Copyright 2020, Wiley‐VCH.

Numerous carbon‐free host materials, such as nitrides, phosphides, and oxides, with superior catalytic capacity for sulfur, strong affinity for Li, and high conductivity, have been proposed to avoid the challenges presented by 3D‐carbon hosts. Manthiram et al. proposed an aerogel based on 3D vanadium nitride (VN) nanowires (**Figure** **8**a) as a bi‐functional host for both electrodes, which improved the performance of LSBs^54^. As shown in Figure 8b,c, the aerogel exhibited a highly porous structure, which enhanced electrolyte infiltration, with highly conductive cross‐linked VN networks, which provided fast electron transport paths. As shown in Figure 8d, the symmetrical cell with VN aerogel–based electrodes exhibited a stronger current response than the symmetrical cell using carbon nanotube (CNT)‐based electrodes, which implies a superior catalytic ability of the cathode. Moreover, compared with pure Li foil, VN–Li showed excellent stability without noticeable fluctuations during the cycling process at 10 mAh cm^−2^ (Figure 8e). The uniform deposition of Li on the VN substrate is illustrated in the scanning electron microscopy (SEM) images after Li plating (Figure 8f) and striping (Figure 8g). These results suggest high catalytic activity of the aerogel toward the sulfur reaction and a strong affinity to Li, which can be attributed to the polar and unsaturated bonds formed between V and N. Thus, LSBs using VN‐nanowire‐aerogel hosts in both electrodes retained a capacity of ≈750 mAh g^−1^ with a high CE (99.6%) after 850 cycles at 4.0 C, a sulfur loading of 4.0 mg cm^−2^, and an E/S ratio of 10 µL mg^−1^.

The host material requires good mechanical properties to withstand the high pressure used during battery assembly. However, it is difficult to achieve a 3D structure that has good mechanical properties using these carbon‐free host materials, especially when large‐scale preparation methods are used. This is due to their poor binding strength compared to the polymer precursors used to prepare carbon materials. It is obviously inappropriate to solve the practical problems by using hosts that cannot be prepared at scale.

## 3 Design of Powder Hosts with Superior Bi‐functionality

Developing bi‐functional powder hosts that can be used to prepare electrodes using a casting method is important because 3D carbon‐free hosts are difficult to prepare using simple methods. The lack of a 3D structure in these materials should be compensated by their superior bi‐functionality and high conductivity, which are directly related to the overall functionality of the materials. Therefore, it is necessary to study methods to improve the bi‐functionality of materials. Below are the existing strategies.

### 3.1 Improving Bi‐functionality via Size Control

Reducing the material size is a common method to provide abundant adsorption and catalytic sites for PSs and Li‐deposition sites. However, small‐sized materials can easily aggregate when used as electrode materials,^[^ ^55^ ^]^ and hence, they often require a substrate that can inhibit agglomeration.

Two‐dimensional (2D) materials, including graphene derivatives,^[^ ^56^, ^57^ ^]^ MXenes,^[^ ^58^ ^]^ and transition‐metal oxides,^[^ ^59^ ^]^ have been extensively explored since the discovery of graphene in 2004. Owing to their large specific surface areas, high electrical conductivities, numerous functional groups, and good mechanical properties, 2D materials are promising substrates for small‐sized materials.^[^ ^60^ ^]^

#### 3.1.1 Nanomaterials

**Figure 9:**
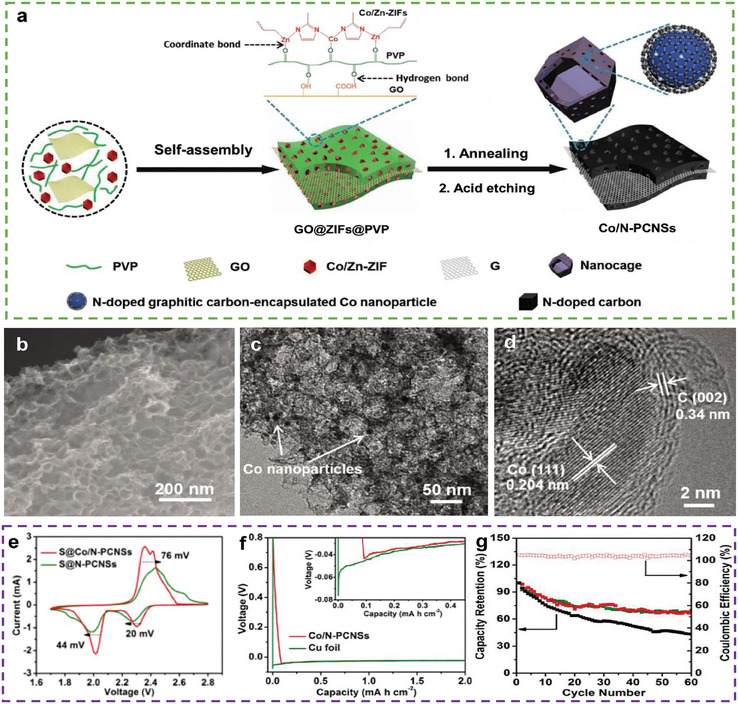
a) Schematic illustration of the synthesis of Co/N‐PCNSs b) SEM image and c,d) TEM image of Co nanoparticles encapsulated in graphitic carbon layers. e) CV curves of S@Co/N‐PCNSs and S@N‐PCNSs cathodes at a scan rate of 0.2 mV s^−1^. f) Voltage profiles of Li plating on Cu foil and Co/N‐PCNSs at 0.5 mA cm^−2^. g) Cycling performance of S@Co/N‐PCNSs cathodes coupled with Li@Co/N‐PCNSs (red), Li foil (green), or Li@Cu (black) anodes at 0.2 C for 60 cycles. (a–g) Reproduced with permission^[^ ^61^ ^]^ Copyright 2018, Wiley‐VCH.

Wu et al. prepared a special 2D cobalt‐embedded nitrogen‐doped porous carbon nanosheet (Co/N‐PCNSs)^[^ ^61^ ^]^ using the process shown in **Figure** **9**a. The large, specific surface area of graphene prevents material agglomeration through adsorption. This, along with its high conductivity, allows an easy linking with the functional groups and even loading of small‐sized functional materials on its surface, ensuring superior bi‐functionality of the loaded material. The SEM (Figure 9b) and transmission electron microscopy (TEM) images (Figure 9c,d) illustrate the uniform distribution of small‐sized Co particles. At the cathode, the increased redox peak intensity (Figure 9e) suggests that the stable graphitic carbon encapsulated–Co nanoparticles can accelerate the conversion kinetics of sulfur. At the anode, the different nucleation overpotentials of Li tablets using Cu foil and Co/N‐PCNSs as collectors are demonstrated in Figure 9f. This indicates a lower plating resistance of the Li/Co/N‐PCNS electrode, which can be attributed to the high‐density lithiophilic N heteroatoms in the carbon frameworks. Moreover, the large surface area reduces the local current density of Li deposition, ensuring uniform Li deposition and contributing to stable cycling performance. The hierarchical structure can provide sufficient space for the large volume change during the charge–discharge of both the cathode and anode. As shown in Figure 9g, S@Co/N‐PCNSs cathodes with a sulfur loading of 0.8–1.0 mg cm^−2^ and an E/S ratio of 20 µL mg^−1^ coupled with Li@Co/N‐PCNSs exhibit a capacity retention of 68% over 60 cycles at 0.2 C.

#### 3.1.2 Quantum Dots

**Figure 10:**
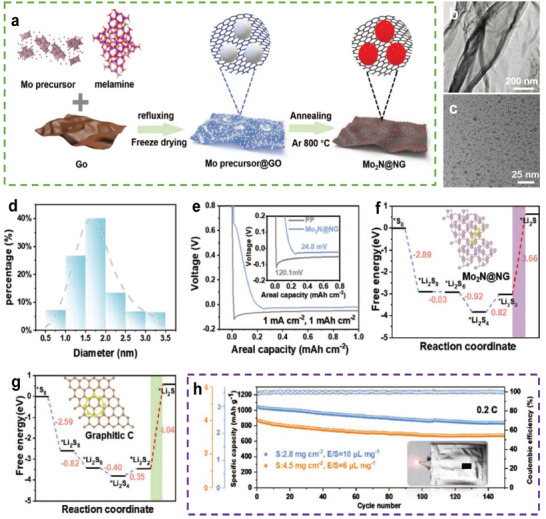
a) Schematic diagrams of the synthetic procedure of Mo_2_N@NG. b) TEM images of Mo_2_N@NG. c) High‐resolution TEM image of Mo_2_N@NG. d) The corresponding particle size distribution pattern. e) Voltage profiles of the lithium plating and stripping process during the first cycle at 1 mA cm^−2^ with a constant capacity of 1 mAh cm^−2^ (Inset: amplified galvanostatic discharge curves). (Free‐energy profiles for the S conversion on the surface of f) Mo_2_N (111) and g) graphitic carbon surface. h) Cycling performance of the pouch cell with Mo_2_N@NG/PP separator under various high‐sulfur loadings. (a–h) Reproduced with permission^[^ ^62^ ^]^ Copyright 2020, American Chemical Society.

Recently, quantum dots have been widely used in numerous fields owing to their abundance of highly active sites, which result from the quantum‐size effect. Some quantum dots can provide abundant adsorption and catalytic sites for PSs and Li‐deposition sites.^[^ ^62^ ^]^ Wu et al. reported a novel sulfiphilic and lithiophilic interlayer of Mo_2_N quantum dot–decorated N‐doped graphene nanosheet (Mo_2_N@NG) as a bi‐functional interlayer for the simultaneous reduction of Li dendrites and shuttle effect in LSBs (**Figure** **10**a).^[^ ^63^ ^]^ As shown in Figure 10b–d, Mo_2_N@NG quantum dots (average size = 2 nm) were evenly loaded onto NG. Figure 10e shows a low Li‐nucleation overpotential (24.8 mV) on Mo_2_N@NG owing to its high interfacial conductivity. The superior catalytic activity of Mo_2_N@NG for the sulfur reaction was confirmed through density functional theoretical (DFT) calculations. As shown in Figure 10f–g, the energy barrier of the rate‐determining step (Li_2_S_2_^*^ reduction to Li_2_S^*^) on Mo_2_N (3.66 eV) is lower than that of graphitic N (≈4.04 eV). Thus, pouch LSBs based on Mo_2_N@NG/PP exhibited 700 mAh g^−1^ (≈80% of the initial capacity) at a sulfur loading of 4.5 mg cm^−2^ and an E/S ratio of 6 µL mg^−1^ after 130 cycles at 0.2 C, demonstrating the potential of these materials for practical applications (Figure 10h).

#### 3.1.3 Monatomic Materials

Monatomic materials are widely used in LSBs because they can selectively and effectively catalyze the electrochemical reactions of sulfur, owing to their large exposed surface area and adjustable electronic structures.^[^ ^64^ ^]^

**Figure 11:**
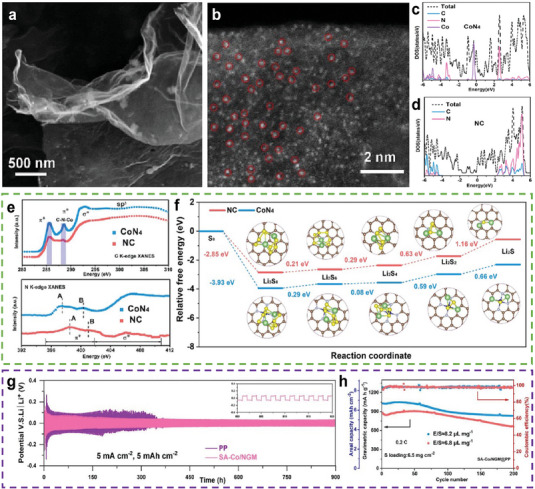
a) Magnified SEM image and b) AC‐HAADF‐STEM image of SA‐Co/NGM. c,d) Calculated density of states for NC and CoN_4_. e) C K‐edge and N K‐edge XANES spectra of NC, CoN_4_. f) Relative free energy for the discharging process from S_8_ to Li_2_S. The optimized structures of the intermediates on the NC and CoN_4_ surfaces are shown in the insets. The brown, white, blue, yellow, and green balls represent C, N, Co, S, and Li atoms, respectively. g) Galvanostatic cycling of Li symmetric cell at a high current density of 5 mA cm^−2^ under a capacity of 5 mAh cm^−2^ based on SA‐Co/ NGM modified separator (inset is the local enlargement of selected cycles) h) Cycling performance of LSBs with SA‐Co/NGM@PP at 0.2 C. (a–h) Reproduced with permission^[^ ^65^ ^]^ Copyright 2023, Elsevier.

Gu et al. modified a separator using Co single atoms (Co–N_4_) loaded onto a nitrogen‐doped graphene mesh (SA‐Co/NGM) (**Figure** **11**a).^[^ ^65^ ^]^ The Co single atoms were uniformly dispersed on the matrix, exhibiting an ultra‐mesh morphology (Figure 11a,b). After introducing the Co single‐atom sites, Co–N_4_ revealed a higher spin state and metallic conductivity with an increase in the density of state at the Fermi level (Figure 11c,d). This indicates a charge transfer phenomenon between the metal and the nitrogen‐doped graphene mesh substrate (Figure 11e). Therefore, SA‐Co/NGM demonstrates effective bi‐functionality. The catalytic activity of Co–N_4_ for sulfur conversion was confirmed using DFT calculations (Figure 11f), which indicated that the energy barrier of the rate‐limiting step for the sulfur reaction on Co–N_4_ was lower than that on other surfaces. Figure 11g shows that a Li symmetrical cell with SA‐Co/NGM@PP exhibited a stable cycling process over 900 h at 5 mA cm^−2^, indicating that the Co–N_4_ sites can homogenize the Li‐ion flux and control Li growth. Thus, LSBs based on SA‐Co/NGM@PP exhibits a capacity of 727.7 mAh g^−1^ (4.73 mAh cm^−2^) at a sulfur loading of 6.5 mg cm^−2^ and an E/S ratio of 6.8 µL mg^−1^ after 200 cycles at 0.2 C (Figure 11h).

Compared to the SAC sites, dual‐atom sites, which contain a pair of atoms linked by chemical bridge bonds, exhibit superior bi‐functionality for LSBs owing to the proximity of the atoms and their unique coordination.

**Figure 12:**
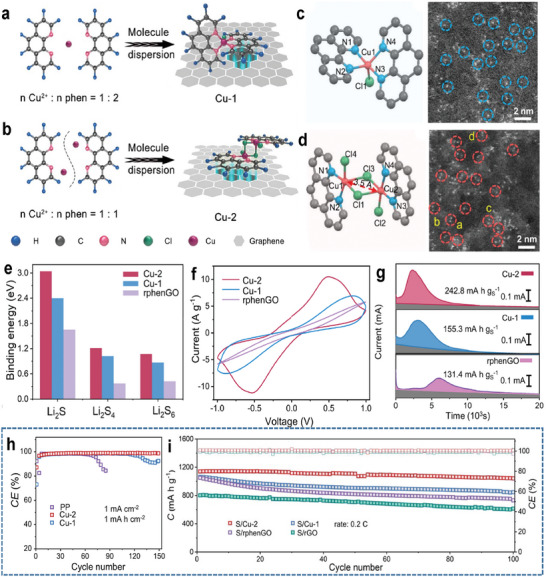
Schematically illustrating the designs of Cu‐1 a) and Cu‐2 b). c,d) Molecular structure and atomic‐resolution HAADF‐STEM images of Cu‐1 c) and Cu‐2 d). e) Binding energies of sulfur species on various substrates. f) CV profiles of Cu‐2, Cu‐1, and rphenGO‐based symmetric cells. g) Potentiostatic discharge curves of Li_2_S_8_/tetraglyme solution on different substrates. h) CE of Cu//Li cells with different separators at 1 mA cm^−2^. i) Cycling stability of the cathodes with different separators at 0.2 C. Reproduced with permission^[^ ^66^ ^]^ Copyright 2024, Nature Publishing Group.

For example, Song et al. synthesized a mononuclear Cu atom host (Cu‐1, **Figure** **12**a) and a homonuclear dual Cu atoms host (Cu‐2, Figure 12b), and then compared their bi‐functional activity for LSBs.^[^ ^66^ ^]^ Different from Cu‐1 (Figure 12c), the molecular structure of Cu‐2 showed that two adjacent copper atoms in Cu‐2 are linked by a pair of symmetrical chlorine (Cl) bridge bonds (Figure 12d). Owing to the proximal copper atoms (with a distance of ≈3.5Å) and their unique coordination, Cu‐2 displayed stronger adsorption of PSs (Figure 12e), faster kinetic of sulfur reaction (Figure 12f,g), and more stable Li plating/stripping process (Figure 12h). As a result, the initial capacity and the cycle performance of LSBs with Cu‐2 are better than that of LSBs with Cu‐1. Pouch LSBs with Cu‐2 can achieve stable cycling for more than 300 cycles, with a capacity decay of 0.11% per cycle at 0.2 C (Figure 12i).

Therefore, reducing the size of the materials is an effective strategy for enhancing their bi‐functionality. However, small‐sized materials, particularly quantum dots and single atoms, are not sufficiently stable during cycles. Moreover, it is difficult to increase the number of quantum dots and single atoms in composite hosts, which is essential for their overall bi‐functionality. Therefore, it is necessary to develop strategies that do not rely heavily on size.

### 3.2 Improving Bi‐functionality through Controlling Electron Transfer

Controlling electron transfer in materials to form various electron acceptor or donor sites is also a common method for improving their bi‐functionality.

#### 3.2.1 Controlling Electron Transfer by Forming Heterostructures

Constructing heterostructures is a common method for enhancing the electron donating and accepting characteristics of materials. Some heterostructured materials exhibit catalytic activity for sulfur and affinity to Li^67^. Moreover, heterostructured materials should have a suitable size, which requires measures to prevent agglomeration. To reduce the complexity of the preparation, a simple strategy involving the preparation of a substrate with a large specific surface area and high conductivity loaded with a small‐sized material is used to construct high‐performance bi‐functional heterostructures. It should be noted that the internal electric field of heterojunctions comprising a graphene substrate and loading material is generally weaker than that of other heterojunctions.^[^ ^68^ ^]^ Therefore, the structures of carbon materials loaded with active materials proposed in several studies have not considered heterojunctions.^[^ ^69^ ^]^

2D transition‐metal carbides, or nitrides (called MXenes), are a new family of 2D materials that exhibit several unique properties. Typically, MXenes are prepared by etching the “A” layer on the laminated MAX phases (M_n+1_AX_n_, *n* = 1–4), in which M represents a transition metal (e.g., Ti, V, Zr, and Nb), A is a IIIA or IVA element (e.g., Al, Ga, and Si), and X represents carbon or nitrogen. After removing A atoms by the etching process, MXenes (general formula: M_n+1_X_n_T_x_) with various surface terminations can be obtained, where “T” refers to different terminations (e.g., –O, –OH, –F, and –Cl).^[^ ^70^ ^]^ MXenes are suitable hosts for sulfur cathodes and Li‐metal anodes owing to their unique structure and characteristics, such as metallic conductivity, high surface area, various terminations, and other characteristics of 2D materials.^[^ ^71^ ^]^ The large specific surface area and various surface terminations of these materials can provide an effective confinement effect, resulting in the uniform loading of functional materials on their surfaces. The MXene substrate and the loaded material easily form heterostructured materials, which have been widely used in several fields owing to their controllable properties compared with those of homogeneous materials. Therefore, MXene‐based heterostructured materials have been proposed as bi‐functional powder hosts for LSBs.

**Figure 13:**
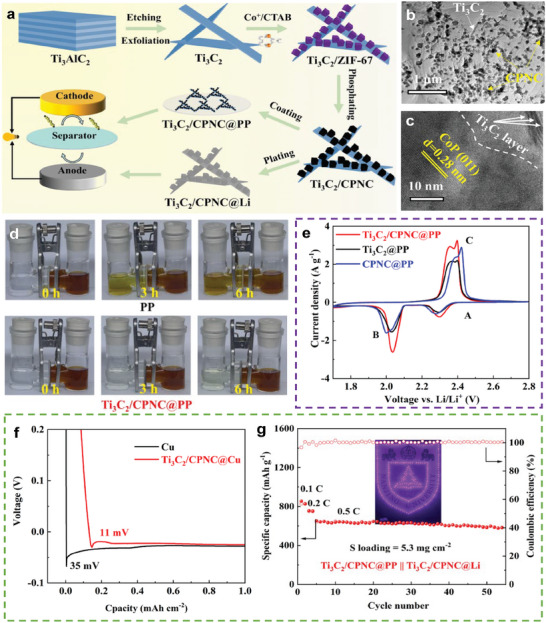
a) Schematic illustration of the synthetic procedures of Ti_3_C_2_/CPNC as an interlayer and Li host for well‐designed LSBs. b,c) TEM images of Ti_3_C_2_/CPNC. d) Penetration experiment with Ti_3_C_2_/CPNC@PP and pure PP separators. e) CV curves of LSBs with different separators at a scanning rate of 0.1 mV S^−1^. f) Discharge curves at 0.5 mA cm^−2^ (1 mAh cm^−2^). g) Cycling performance of LSBs with Ti_3_C_2_/CPNC@PP and Ti_3_C_2_/CPNC@Li. (a–g) Reproduced with permission^[^ ^72^ ^]^ Copyright 2022, Elsevier.

Meng et al. reported a bi‐functional host comprising an MXene‐based heterostructure in which CoP nanocages (CPNC) were intercalated into Ti_3_C_2_T_x_ (Ti_3_C_2_) nanosheets (**Figure** **13**a).^[^ ^72^ ^]^ As shown in Figure 13b,c, Ti_3_C_2_ nanosheets can prevent the agglomeration of CoP nanocages and CoP also suppresses the recombination of Ti_3_C_2_ nanosheets, thus providing abundant active sites. Ti_3_C_2_/CPNC exhibited superior adsorption capacity toward PSs (Figure 13d) owing to its numerous bonds, such as N–S, P–S, and Ti–S. Moreover, electrons could be transferred from Li_2_Sn to the Co atoms, accelerating the transformation of sulfur. This was confirmed by the CV curves in Figure 13e. In addition, Ti_3_C_2_/CPNC reduces the Li nucleation barrier owing to the superior conductivity and lithiophilicity of Ti_3_C_2_ (Figure 13f). Therefore, a Ti_3_C_2_/CPNC@PP|| Ti_3_C_2_/CPNC@Li full cell exhibited an initial capacity of 1280.1 mAh g^−1^ at 0.1 C and a capacity of 520 mAh g^−1^ after 50 cycles at 0.5 C under the conditions of a sulfur loading = 5.3 mg cm^−2^, N/P ratio = 1.7:1, and E/S ratio = 20 µL mg^−1^ (Figure 13g).

#### 3.2.2 Controlling Electron Transfer via Strong Coordination Bonds

Electron acceptor and donor sites are found in some materials with strong coordination bonds, such as metal–organic frameworks (MOFs, also called porous crystalline polymers), which comprise metal centers (clusters) and organic ligands (organic linkers) connected through strong coordination bonds. MOFs exhibit a large surface area and porous structures. Thus, MOF composites and MOF‐derived materials are extensively used in the design of bi‐functional hosts for LSBs.^[^ ^73^ ^]^

**Figure 14:**
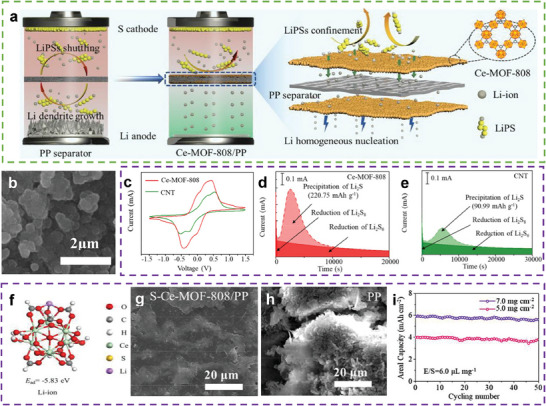
a) Schematic illustrations of the advantages of the Ce‐MOF‐808/PP separator. b) SEM image of Ce‐MOF‐808/PP separator. c) CV profiles of symmetrical cells at 5 mV s^−1^. Chronoamperometry curves of a Li_2_S_8_/tetraglyme solution at 2.05 V on the surfaces of d) Ce‐MOF‐808 and e) CNT cathodes. f) The optimized adsorption configurations and corresponding adsorption energies of Li‐ion on Ce‐MOF‐808. SEM morphologies of the cycled Li‐metal anode in Li symmetric cells with g) S‐Ce‐MOF‐808/PP and h) PP separator. i) Cycle performance at 0.2 C of LSBs with Ce‐MOF‐808/PP. (a–i) Reproduced with permission^[^ ^74^ ^]^ Copyright 2022, Elsevier.

Li et al. synthesized cerium‐based MOF‐808 (Ce‐MOF‐808) membranes on both sides of a commercial polypropylene (PP) separator to simultaneously reduce the shuttle effect and formation of Li dendrites (**Figure** **14**a).^[^ ^74^ ^]^ The SEM image in Figure 14b shows that the surface of the PP separator is fully covered by Ce‐MOF‐808. The strong affinity of the material for PSs and Li ions was confirmed based on DFT calculations (Figure 14c). The catalytic activity for PSs conversion was confirmed based on the CV curve (Figure 14d), which indicated that Ce‐MOF‐808 exhibited the largest response current. The potentiostatic discharge curve of Ce‐MOF‐808 (Figure 14e) shows an earlier deposition peak and a considerably larger area than that of CNT (Figure 14f), indicating facile nucleation kinetics of Li_2_S on Ce‐MOF‐808. Owing to the strong adsorption of Li ions on S‐Ce‐MOF‐808/PP (Figure 14g), even Li deposition can be achieved on its surface (Figure 14h,i). Thus, the LSBs with the Ce‐MOF‐808/PP separator exhibited an initial capacity of 954.7 mAh g^−1^ and a capacity retention of 87% (834.3 mAh g^−1^) after 500 cycles at 0.2 C, even at a high‐sulfur loading (7.0 mg cm^−2^) and a low E/S ratio (6.0 µL mg^−1^) (Figure 14f).

Covalent organic frameworks (COFs), a promising class of porous crystalline polymers featuring regular porous structures and tunable chemical/electronic environments, are another class of materials with strong coordination bonds. Their ordered pores and abundant polar functional sites, resulting from tunable electronic environments, can help to effectively suppress the “shuttle effect” of PSs through physical‐ and chemical‐ adsorption. Meanwhile, Li^+^ transportation can be regulated by COFs, owing to their modifiable nanochannel walls of COFs which can promote host‐guest interactions. Therefore, COFs also exhibit a large potential for application in LSBs. However, due to the absence of efficient catalytic sites within COFs for sulfur reactions, it is essential to incorporate catalytic sites into COFs when they serve as bi‐functional hosts in practical LSBs.

**Figure 15:**
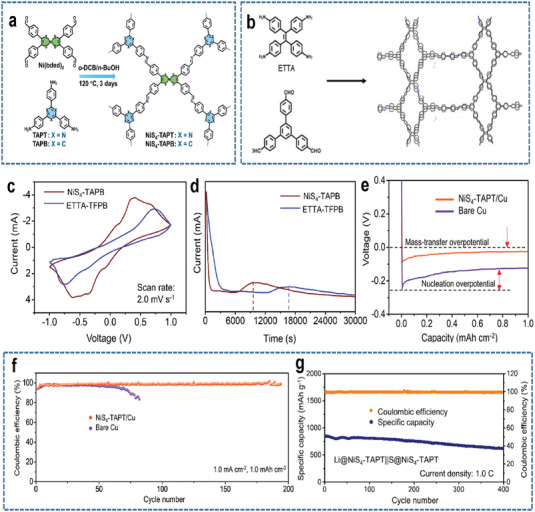
Schematic diagram of the synthesis processes of NiS_4_‐TAPT a) and ETTA‐TAPB b). c) CV curves of symmetric batteries based on Li_2_S_6_ catholyte within different host materials. d) Potentiostatic discharge profiles of asymmetric cells based on Li_2_S_8_ catholyte within different host materials at 2.05 V. e) Voltage profiles during the initial Li plating process on different collectors. f) Coulombic efficiency of Li||NiS_4_‐TAPT/Cu and Li||bare Cu half batteries. g) Long‐term cycling performance of LSBs using NiS_4_‐TAPT as bi‐functional hosts. Reproduced with permission^[^ ^75^ ^]^ Copyright 2024, American Chemical Society.

For example, Zuo et al. synthesized a novel metal‐coordinated 3D porous COF named NiS_4_‐TAPT, which features uniform Ni‐bis (dithiolene) moieties and rich N sites (**Figure** **15**a).^[^ ^75^ ^]^ Compared with a COF named ETTA‐TFPB (Figure 15b, which is similar isostructural to NiS_4_‐TAPT), NiS_4_‐TAPT exhibited stronger catalytic activity for sulfur reaction (Figure 15c,d), validating the necessity of Ni centers. As shown in Figure 15e,f, the Li plating/stripping process on NiS_4_‐TAPT/Cu is more facile and stable than that on Cu. This is because the high porosity and uniform pore size of the NiS_4_‐TAPT can enhance the homogeneous distribution of Li‐ion flux and fast Li‐ion transport. As a result, the NiS_4_‐TAPT as bi‐functional hosts enable the LSBs to exhibit an initial capacity of ≈800 mAh g^−1^ and a capacity retention of 73% after 400 cycles (Figure 15g). However, the sulfur loading (≈2 mg cm^−2^) and E/S ratio (≈15 µL mg^−2^) in the above LSBs are still far from practical application, demonstrating the necessity of further enhancing the catalytic activity of COFs.

Efficient bi‐functional hosts can be manufactured through controlling electron transfer. However, they are generally heavier than other bi‐functional hosts (**Table** **1** ^[^ ^38^, ^51^, ^65^, ^72^, ^74^, ^76^, ^77^, ^78^, ^79^, ^80^ ^]^), resulting in a lower actual specific capacity because of the high mass of the hosts, which considerably affects the actual energy density of LSBs.

**Table 1:** The performances of recently reported LSBs based on hosts with unique structures.

Classification	Cathode	S loading [mg cm^−2^]	E/S [µL mg^−1^]	S content	Capacity _based on S_ [mAh g^−1^]	Capacity_based on S + host_ [mAh g^−1^]	rate	Refs
Heterostructures or MOF	S/ZnSe‐CoSe_2_@NC	6.08	4.1	56%	≈600	≈336	0.2 C	[30]
	Carbon black/S+PVDF+NMP (B/2D MOF‐Co)	7.8	5.1	56%	≈641	≈358	0.5 C	[64]
	AP/S+PVDF+acetylene black (Ti_3_C_2_/CPNC@PP)	5.3	20	21%	1280.1	268.8	0.1 C	[61]
	Nb_4_N_5_–Nb_2_O_5_/S	6.9	5.1	64%	≈724	≈463.4	0.3 C	[65]
	CNT/S+PVDF+NMP (S‐Ce‐MOF‐808/PP)	7.0	6.0	55%	852.8	463.6	0.2 C	[63]
Carbon composite materials	S/CoSe@C	6.2	4.5	70%	860	602	0.2 C	[43]
	CNF with polar oxygen groups	6	1.7	73%	900	657	0.2 C	[66]
	3D‐printed Co/Co‐N@nitrogen‐doped porous carbon fibers	7.1	6.33	58.5%	901	527.1	0.2 C	[67]
	CNT/S+PVDF+SuperP (SA‐Co/nitrogen‐doped graphene mesh @PP)	6.5	6.8	64%	727.7	465.7	0.2 C	[57]
	ZnN_4_‐ nitrogen‐doped graphene	7.2	3.7	60%	953.4	527.04	0.1 C	[68]

## 4 Design of Bi‐functional Electrolytes

### 4.1 Formation of Stable Interfaces on Both Electrodes

**Figure 16:**
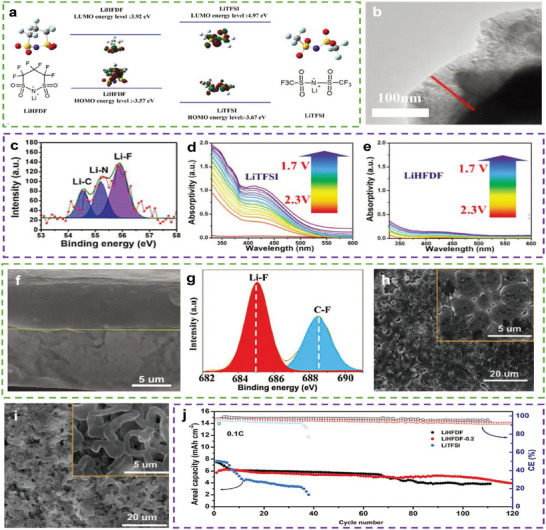
a) The molecular structure, molecular model, and HOMO and LUMO energies of LiTFSI and LiHFDF. b) TEM image and c) Li 1s XPS of cathode using LiHFDF electrolyte after 5 cycles. In situ UV spectrum of quartz vessel LSBs system with d) LiTFSI electrolyte and e) LiHFDF electrolyte during discharge processes at the current of 0.2 C.f) Cross–sectional SEM and g) F 1s XPS of symmetric Li anode using LiHFDF electrolyte at the current density of 0.5 mA cm^−2^ after 2 cycles. SEM of Li anode obtained in symmetric Li cells using h) LiHFDF electrolyte and i) LiTFSI electrolyte after 20 cycles. j) Cycling performance of LSBs with LiHFDF, LiHFDF‐0.2, and LiTFSI electrolyte at 0.1 C. (a–j) Reproduced with permission^[^ ^82^ ^]^ Copyright 2020, Wiley‐VCH.

Several studies have reported that the shuttle effect and formation of Li dendrites can be prevented by generating dense and stable interfaces between the electrolyte and electrodes.^[^ ^81^ ^]^ Therefore, using additives to form dense and stable interfaces on both electrodes is a promising “two‐in‐one” strategy. Xu et al. added a novel Li salt called lithium 1, 1, 2, 2, 3, 3‐hexafluoropropane‐1, 3‐disulfonimide (LiHFDF) to an LSB electrolyte.^[^ ^82^ ^]^ Owing to the low lowest unoccupied molecular orbital (LUMO) and highest occupied molecular orbital (HOMO) of LiHFDF (**Figure** **16**a), it can react before lithium bis((trifluoromethyl)sulfonyl)azanide (LiTFSI) to form the required interfaces. As shown in Figure 16b,c, after five cycles, LiHFDF can form a cathode–electrolyte interface (CEI), which mainly comprises Li–F, Li–N, and Li–C, on the surface of the cathode. As shown in Figure 16d,e, bare PSs are soluble in the LiHFDF electrolyte, indicating that this CEI can effectively inhibit the diffusion of PSs from the sulfur cathode to participate in the “shuttle effect”. Moreover, a LiF‐rich SEI can be found on the Li‐metal anode after two cycles (Figure 16f,g). As shown in Figure 16h,i, the surface of the cycled Li‐metal with the LiHFDF electrolyte was smoother than that with LiTFSI, suggesting that this SEI can suppress the growth of Li dendrites. Owing to their bi‐functionality, LSBs based on LiHFDF exhibited an initial capacity of 896 mAh g^−1^ and a retained capacity of 51% after 110 cycles at a sulfur loading of 8.36 mg cm^−2^ and an E/S ratio of 15 µL mg^−1^ (Figure 16j).

However, the CEI formation process, using these bi‐functional electrolyte additives, is completed after a certain number of cycles, during which numerous PSs dissolve out of the cathodes. These PSs are difficult to recover from the CEI. Moreover, because the process of CEI formation is uncontrollable, the sulfur reaction sites can be easily covered, which affects sulfur utilization.^[^ ^83^ ^]^

While the artificial hybrid interphase, typically employed to inhibit Li dendrite formation, can also mitigate side reactions between PSs and the Li metal anode (a major contributor to active sulfur loss) by preventing their contact, it falls short of serving the needs of practical LSBs as an effective bi‐functional material. For example, Xiong et al. developed pouch LSBs utilizing a Li metal anode coated with an artificial hybrid interphase, which consisted of Li_3_Sb and LiF.^[^ ^84^ ^]^ Under practical conditions, including a high sulfur loading of 6 mg cm^−2^ and a low E/S ratio of 3 µL mg^−1^, the coulombic efficiency of pouch LSBs becomes low and fluctuates after just 30 cycles. The failure of this strategy may be caused by the following reasons: 1) PSs can still shuttle and then be adsorbed on electron‐inaccessible sites (such as the separator, SEI, and so on); 2) artificial layers are incapable of completely preventing contact between PSs and Li metal.

Meanwhile, numerous investigations have demonstrated that high‐performance LSBs under unpractical conditions can be fabricated based on electrolytes designed solely for sulfur cathodes. However, the unmodified Li‐metal anodes lead to continuous consumption of the available electrolyte and Li metal, which can easily affect the performance of LSBs with low E/S and N/P ratios. For example, Huang et al. introduced a selenium‐based additive (diphenyl diselenide, DPDSe) into the electrolyte, which is proposed to accelerate the sulfur redox kinetics as a redox mediator.^[^ ^85^ ^]^ The modified electrolyte enabled the LSBs with high E/S (15.7 µL mg^−1^) and N/P (4.5) ratios to show a low cyclic decay rate of ≈0.091%. In contrast, under low E/S (6.8 µL mg^−1^) and N/P (1.1) ratios, the LSBs utilizing this modified electrolyte displayed a significantly higher cyclic decay rate of ≈0.33%. These observations underscore the necessity of bi‐function design.

### 4.2 Controlling the Migration of Anions and Distribution of Li^+^

Numerous studies have reported that solid‐state electrolytes (SSEs) can be used to achieve “two‐in‐one” strategies. Inorganic solid electrolytes (ISEs), which include oxides, sulfides, halides, hydroborons, and so on, possess high ionic conductivity (≈10^−3^ S cm^−1^), good mechanical strength, and insoluble properties with PSs. These characteristics make them potentially bi‐functional electrolytes for LSBs. However, the sulfur reaction process in ISEs (which does not involve the dissolution of PSs), coupled with the inadequate interfacial contact between the ISEs and the sulfur cathode, significantly hampers the sulfur utilization. Additionally, the side reactions between ISEs and the Li metal anode exert a detrimental influence on the cycling performance of LSBs. Therefore, addressing the aforementioned issues is imperative for effectively utilizing ISEs as bi‐functional electrolytes for LSBs.

**Figure 17:**
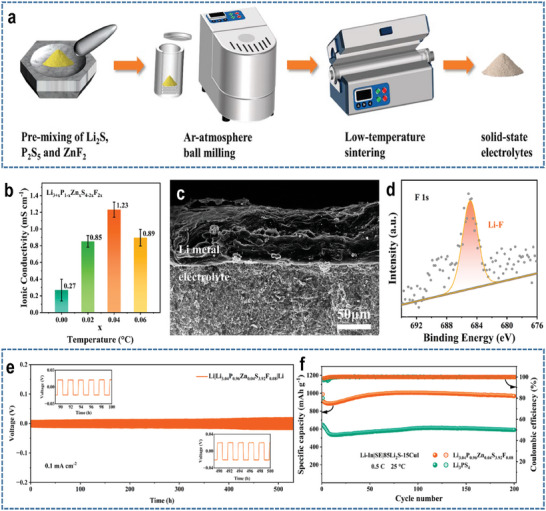
a) Schematic diagram of Li_3+x_P_1−x_Zn_x_S_4−2x_F_2x_ preparation. b) Impedance plots of Li_3+x_P_1−x_Zn_x_S_4−2x_F_2x_ (x = 0, 0.02, 0.04, and 0.06). c) Cross–section SEM images of the Li|Li_3.04_P_0.96_Zn_0.04_S_3.92_F_0.08_|Li symmetric cell after cycling 550 h at 0.1 mA cm^−2^. d) XPS spectra of F 1s at the Li metal surface after Li plating/stripping in the symmetric cell. e) Li|Li_3.04_P_0.96_Zn_0.04_S_3.92_F_0.08_|Li symmetric cell at a constant current density of 0.1 mA cm^−2^. f) Cycling performance of Li‐In|Li_3.04_P_0.96_Zn_0.04_S_3.92_F_0.08_|85Li_2_S‐15CuI cell at 0.5 C. Reproduced with permission^[^ ^86^ ^]^ Copyright 2024, American Chemical Society.

For example, Wu et al. successfully prepared Zn, F co‐doping Li_3_PS_4_ (Li_3.04_P_0.96_Zn_0.04_S_3.92_F_0.08_) as a solid‐state electrolyte for LSBs (**Figure** **17**a).^[^ ^86^ ^]^ Owing to the Zn, F co‐doping, Li_3.04_P_0.96_Zn_0.04_S_3.92_F_0.08_ exhibited high ionic conductivity (Figure 17b) and could form a stable LiF interface with Li metal anode (Figure 17c,d). Therefore, the symmetric Li|Li_3.04_P_0.96_Zn_0.04_S_3.92_F_0.08_|Li cell displayed stable cycling for more than 550 h (Figure 17e). Moreover, activated 85Li_2_S‐15Cul was employed as the cathode active material to enhance sulfur utilization, and Li‐In alloy was applied Li‐In alloy to further mitigate the problem of side reactions. As a result, 85Li_2_S‐15CuI|Li_3.04_P_0.96_Zn_0.04_S_3.92_F_0.08_|Li‐In full cell exhibited a high initial capacity (≈900 mAh g^−1^) and stable cycle performance (Figure 17f). However, the sulfur content and areal loading in these LSBs are ≈42% and 0.85 mg cm⁻^2^, respectively, which are significantly below the requirements for practical applications.

To address the above issues, flexible solid‐polymer electrolytes (SPEs) with soluble properties of PSs are introduced in LSBs. Meanwhile, owing to their strong interaction with PSs and more adjustable ion distribution compared with those of liquid electrolytes (LEs), SPEs can slow down the shuttle of PSs and the growth rate of dendrites.

**Figure 18:**
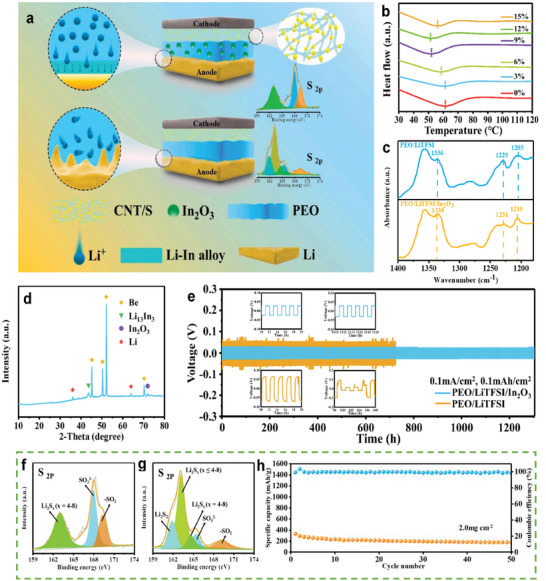
a) Schematic illustration of the mechanism of In_2_O_3_ enhances the performance of PEO‐based solid‐state LSBs. b) DSC profiles of PEO/LiTFSI SPEs with different content of In_2_O_3_ (0%, 3%, 6%, 9%, 12% and 15%). c) FT‐IR spectrum of the PEO/LiTFSI, PEO/LiTFSI/In_2_O_3_ at 1400–1180 cm^−1^. d) The XRD patterns of alloy‐protected Li metal anode after cycling. e) Voltage profiles of Li symmetric cells that cycle at a current density of 0.1 mA cm^−2^ and a capacity of 0.1 mAh cm^−2^. f) The XPS of Li metal anodes obtained in S@C/PEO/LiTFSI/In_2_O_3_/Li and g) S@C/PEO/LiTFSI/Li cells after five cycles. h) Cycling performance of LSBs assembled with the PEO/LiTFSI/In_2_O_3_ SPE at 60 °C and 1 C with a sulfur loading of 2.0 mg cm^−2^ per cell. (a–h) Reproduced with permission^[^ ^87^ ^]^ Copyright 2022, Elsevier.

Min et al. combined polyethylene oxide (PEO) with nano In_2_O_3_ as a multifunctional nanofiller to suppress the growth of Li and reduce the shuttle effect of PSs (**Figure** **18**a).^[^ ^87^ ^]^ Nano‐In_2_O_3_ can reduce the crystallinity of PEO (Figure 18b) and bond with TFSI^−^, promoting the dissociation of Li salts (Figure 18c). Therefore, nano‐In_2_O_3_ can improve the ionic conductivity of SPEs, thereby enhancing the initial capacity of LSBs. During cycling, a Li–In alloy (Li_13_In_3_) layer is formed at the interface between SPEs and the Li‐metal anode (Figure 18d). Owing to the higher bulk diffusion coefficient of Li_13_In_3_ (10^−8^–10^−6^ cm^2^ s^−1^) compared to that of Li‐metal (5.69 × 10^−11^ cm^2^ s^−1^), this Li–In alloy layer can achieve dendrite‐free and stable Li plating (Figure 18e). In_2_O_3_ can also reduce the shuttle effect owing to its higher adsorption capacity toward PSs compared to that of PEO. This is confirmed by Figure 18f,g, which indicates that the number of PSs on the anode with PEO/LiTFSI/In_2_O_3_ is larger than those on the anodes with PEO/LiTFSI. Therefore, at a sulfur loading of 2.0 mg cm^−2^, the LSBs based on PEO/LiTFSI/In_2_O_3_ SPE exhibited an initial capacity of ≈400 mAh g^−1^ and a steady cycling process within 50 cycles at 1 C and 60 °C (Figure 18h).

However, the research of SSEs for LSBs is still in its initial stage. As shown in **Table** **2**,^[^ ^88^, ^89^, ^90^, ^91^, ^92^, ^93^, ^94^, ^95^, ^96^, ^97^, ^98^, ^99^, ^100^, ^101^ ^]^ the sulfur content in the composites and the sulfur loading used in LSBs with SSEs are considerably lower than those used in LSBs with LEs, indicating that there are still many challenges have yet to be concerned in this area.

**Table 2:** The performances of recently reported solid‐state LSBs based on SPEs.

serial number	Solid electrolyte	Sulfur cathode	sulfur loading/ mg cm^−2^	Sulfur content in composite/cathode	Capacity based on sulfur/mAh g^−1^	Areal capacity [mAh cm^−2^]	Capacity based on cathode (without calculating the collector)	Specific capacity [mAh g^−1^] and retention after cycles	Rate	Temperature	Refs
1	PEO/LiTFSI/HNT	PANI@S/C	0.8	38.6%/30.1%	1350	1.08	406.35	766 and 56.74% after 100 cycles	0.1 C	25 °C	[73]
2	BN‐PEO‐PVDF /LiTFSI	S/C	0.8–1.0	90%/40%	1200	1.08–1.2	480	790 and 65.83% after 50 cycles	0.1 C	70 °C	[74]
3	PEO/LiTFSI/P_2_S_5_	S/C	0.5	70%/42%	1049	0.52	440.58	500 and 47.66% after 350 cycles	0.1 C	60 °C	[75]
4	PEO/LiTFSI/HP@TFSI	S/C	0.8	60%/54%	1400	1.12	756	1000 and 71.4% after 50 cycles	0.1 C	60 °C	[76]
5	PEO/LiTFSI/LATP	S/C	2.0	60%/42%	750	1.5	315	628.5 and 83.8% after 320 cycles	0.05 C	35 °C	[77]
6	PEO/PVDF/LiTFSI/BN	S/C	0.6	72.7%/40%	967	0.58	386.8	768 and 79.42% after 100 cycles	0.2 C	55 °C	[78]
7	PEO/LLZO/LiClO_4_	S/C/Li_7_La_3_Zr_2_O_12_ (LLZO)	0.54	64%/49%	≈1040	0.56	665.6	≈900 and 86.54% after 200 cycles	0.05 C	37 °C	[79]
8	PEO/Li_7_P_3_S_11_(LPS)/LiClO_4_	S/CB/LPS	2.0	50%/25%	826	1.652	206.5	394 and 47.7% after 60 cycles	0.05 C	25 °C	[80]
9	PEO/LiTFSI/LLZTO	S/Super P	0.41	78%/70%	1286	0.527	900.2	200 and 15.55% after 50 cycles	0.05 C	65 °C	[81]
10	PEO/LiTFSI /γ‐LiAlO_2_	S/AB	1.5	26.7%//24%	425	0.637	102	184 and 40.71% after 50 cycles	0.1 C	75 °C	[82]
11	PVDF/LiTFSI/BPSO/CA	S/MWCNT/CB	2–3	80%/40%	1493	2.986–4.479	597.2	490 and 32.82% after 80 cycles	1.0 C	25 °C	[83]
12	PEO/LiTFSI/Li_10_SnP_2_S_12_(LSPS)	S/AB	0.5	72.7%/40%	562	0.281	224.8	518 and 92.17% after 150 cycles	0.2 C	60 °C	[84]
13	PEO/LiTFSI/MMT	S/PAN/Mg_0.6_Ni_0.4_O	1.2	76.1%/41.8%	998	1.1976	548.9	634 and 63.53% after 100 cycles	0.1 C	60 °C	[85]
14	PEO/LiTFSI/nanometer alumina	S/C	2.0	50%/25%	825	1.65	206.25	630 and 76.36% after 60 cycles	0.05 C	55 °C	[86]

## 5 Separators Modified by Two Single‐Functional Materials

Single‐functional materials that affect only sulfur cathodes or Li‐metal anodes have been widely studied. Therefore, combining two single‐functional materials with no synergistic effect is the easiest strategy to improve the performance of sulfur cathodes and Li‐metal anodes, and it does not require a complex design. To avoid vast useless functional materials in both sulfur cathodes and Li metal anodes, these single‐functional materials were usually loaded on the separator.

It should be noted that this strategy is certainly worth discussing. It is true that this strategy does not significantly simplify the preparation process and does not reduce the amount of inactive materials. However, this method does not require a complex design and is compatible with existing industrial equipment because modifying separators by coating them with functional materials, such as ceramic and aramid, is the conventional technology used in the industry.

**Figure 19:**
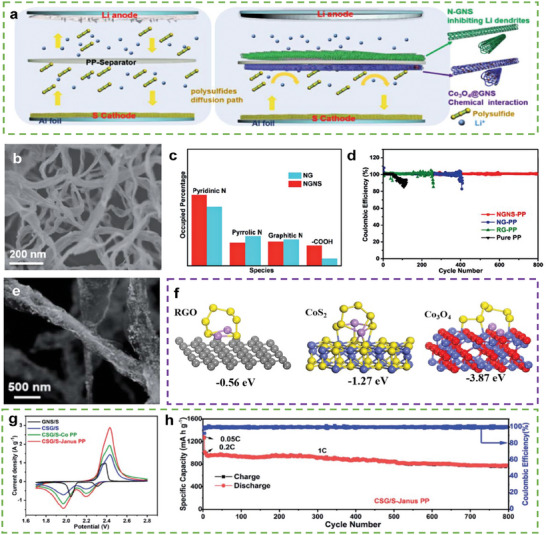
a) Schematic illustrations of LSBs without (left) and with interlayers (right). SEM images of b) NGNS. c) Comparison of different element contents in NG and NGNS. d) Coulombic efficiency comparison of Li–Cu cells with different separators at a current density of 1 mA cm^−2^ and a capacity of 1 mA h cm^−2^. e) SEM of CoS_2_@GNS. f) Adsorption configurations and energies of L_2_S_6_ on RGO, Co_3_O_4_, and CoS_2_‐based compounds. g) CV profiles of symmetrical cells at 0.1C. h) Long‐life cycling stability and corresponding coulombic efficiency of CGS/S‐Janus PP were obtained at the current density of 1C. (a–h) Reproduced with permission^[^ ^102^ ^]^ Copyright 2022, Royal Society of Chemistry.

Li et al. designed a bi‐functional Janus polypropylene (Janus PP) separator by loading different single‐functional materials on its two sides.^[^ ^102^ ^]^ As shown in **Figure** **19**a, N‐doped graphene nanoscrolls (NGNSs) were coated on the anode side of the separator, and GNS‐wrapped Co_3_O_4_ (Co_3_O_4_@GNS) was coated on the cathode side. NGNSs (fiber diameter = 80–100 nm, Figure 19b) have numerous pyridinic N and –COOH, which are lithophilic sites for Li deposition (Figure 19c). Therefore, the Li‐metal anode with NGNS‐PP retained a CE of ≈100% after 800 cycles (Figure 19d). As shown in Figure 19e, the Co_3_O_4_ particles were uniformly distributed on the graphene nanofibers, reducing the effect of their low conductivity on the battery's performance. As shown in Figure 19f, Co_3_O_4_ was used to modify the cathode side of the separator owing to its stronger affinity toward Li_2_S_6_ (3.87 eV) than that of graphene (0.56 eV) and CoS_2_ (1.27 eV). Therefore, the Co_3_O_4_@GNS layer on the separator can accelerate sulfur transformation (Figure 19g). Thus, CSG/S‐Janus PP exhibited an initial capacity of 950 mAh g^−1^ and maintained 805 mAh g^−1^ (a retention of 84.7%) after 800 cycles at 1.0 C at a sulfur loading of 2.7 mg cm^−2^ (Figure 19h).

## 6 Conclusions

**Figure 20:**
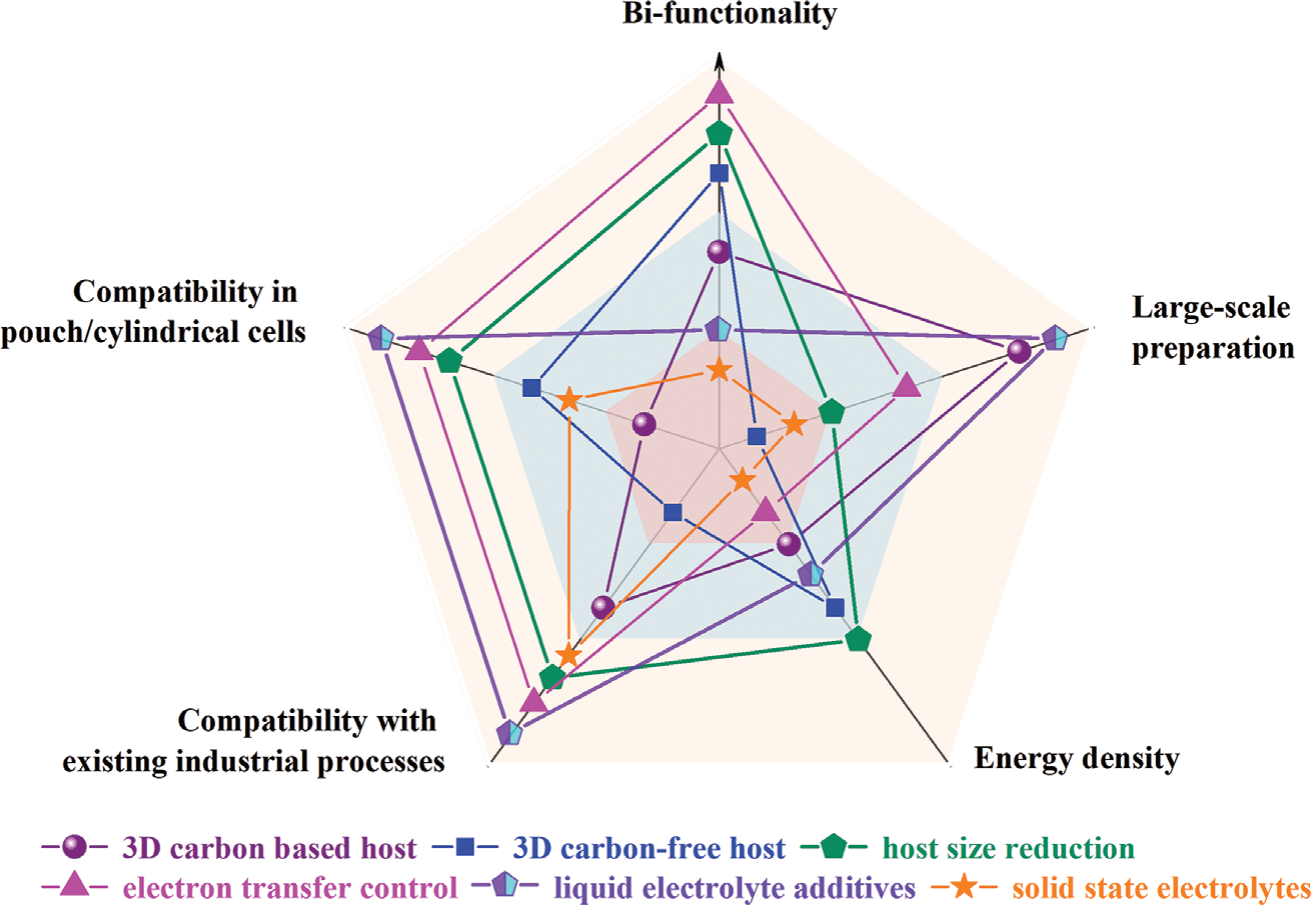
Radar map of the comprehensive evaluation of common strategies.

In this review, we provide a systematic review of the “two‐in‐one” strategies for simultaneously optimizing the sulfur cathode and Li‐metal anode of LSBs. These strategies are categorized based on their design idea, and their advantages and disadvantages are discussed. Despite the current development of “two‐in‐one” strategies, LSBs designed based on them still do not fulfill the requirements of practical applications. Based on a comprehensive evaluation of existing strategies (**Figure** **20** and **Table** **3**), the following challenges should still be overcome:

1. Optimization of 3D hosts. Structural improvements in 3D hosts, such as tunable electron conduction and easy penetration by electrolytes, can reduce the performance requirements of bi‐functional materials for achieving LSBs with high performance under practical conditions. However, in order to reduce agglomeration, the bi‐functionality of materials loaded on 3D hosts (lack of confinement effect) are generally limited, resulting in a high invalid mass. In addition, 3D hosts cannot be easily manufactured on a large scale. The electrospin hosts are brittle after annealing, which is unsuitable for large‐capacity pouch cells or cylindrical batteries. Other 3D hosts, particularly, array structures and suction filtration structures, still cannot be produced on a large scale. Moreover, the preparation of electrodes based on 3D hosts is not compatible with existing industrial processes, increasing the cost of the scaling‐up process. Therefore, how to optimize the bi‐functionality of the 3D carbon‐based host (as a lightweight host with large‐scale preparation methods) without significantly affecting its weight is one promising research direction.
2. Powder hosts with superior bi‐functionality. Materials with superior bi‐functionality can replace 3D hosts, and the preparation of electrodes based on these materials is compatible with existing industrial processes. However, to inhibit the agglomeration of small‐size materials (nanomaterials and quantum dots), these materials typically involve complex preparation processes, and their uniformity when prepared on a large scale cannot be guaranteed. Heterojunction and MOFs materials are generally heavier, which limits the energy density of LSBs. In addition, they require more expensive materials compared to 3D carbon hosts. Market estimates peg the material prices of graphene and MXenes are ≈100 and 2500 US$ Kg^−1^, respectively.^[^ ^103^, ^104^ ^]^ Therefore, how to simply prepare lightweight powder bi‐functional materials without affecting their bi‐functionality is a promising research direction.
3. Bi‐functional electrolytes. Bi‐functional electrolytes based on film‐forming additives do not involve a replication preparation process, and their preparation processes are compatible with existing industrial systems. However, the film‐forming process is difficult to control, limiting the dual functionality of these electrolytes. In addition, this strategy is only applicable to some cathode host structures, including hollow and porous structures. Using SSEs is a fundamental strategy for solving the safety problem of LSBs. However, they exhibit poor functionality for sulfur cathode and low ionic conductivity. Moreover, their preparation and preservation processes have high environmental requirements, i.e., low water and oxygen values, are not suitable for large‐scale application. Therefore, how to improve the performance of SSEs‐based LSBs without compromising their security is a worthy research direction.

**Table 3:** The comprehensive evaluation of common strategies.

Strategies/Evaluations	3D hosts		Powder hosts		Novel electrolytes	
	3D carbon‐based hosts	3D carbon‐free hosts	Host sizes reduction	Electron transfer control	Liquid electrolyte additives	Solid state electrolytes
Bi‐functionality	Medium	High	High	High	Low	Low
Large‐scale preparation	High	Low	Low	Medium	High	Low
Energy density	Low	Medium	Medium	Low	Medium	Low
Compatibility with existing industrial processes Compatibility in pouch/ cylindrical cells	Medium	Low	High	High	High	High
	Low	Medium	High	High	High	High

To address these key challenges, we proposed some possible future directions, which have not received much attention but are worthy of study.

1. Introduction of abundant and efficient bi‐functional edge‐doping sites on carbon nanofibers (CNFs). CNFs are 3D hosts that can be prepared on a large scale using electrospinning. Edge doping can enhance the bi‐functionality of CNF substrates and prevent the mass increase of inactive materials, which occurs when loading common metal‐based bi‐functional materials on 3D hosts. Currently, carbon powder materials are the only materials where edge‐doping sites are introduced. This is achieved by increasing their specific surface area or introducing numerous micropores into the material. However, the above schemes can affect the infiltration electrolyte under low E/S ratios. Up to now, the construction of edge‐doping sites on CNFs without affecting their electrolyte wettability has not been extensively studied.
2. Bi‐functional hosts with abundant monatoms. Single atoms are the primary choice for constructing efficient and lightweight bi‐functional hosts. However, the high loads of single atoms required to achieve this still represent a considerable challenge. Moreover, the bi‐functionality of single atoms has not been clearly studied, which hinders the efficient selection of atoms. Therefore, these challenges should be explored.
3. Development of SPEs suitable for LSBs. At present, research on SPE‐based LSBs (i.e., containing SPE and a sulfur cathode) has not received considerable attention. The improvement strategies in this area remain these strategies used in SPE‐based lithium metal batteries (without sulfur cathode) and LSBs using liquid electrolytes (without SPE). Since these strategies do not consider the interaction of SPEs and the sulfur‐positive electrode, the performance of SPE‐based LSBs remains considerably lower than that of LE‐based LSBs. Therefore, the corresponding solutions in this field are worth studying.

Nevertheless, we believe that the practical development of LSBs can be better promoted by devoting more and more attention to developing “two‐in‐one” strategies.

## Conflict of Interest

There authors declare no conflict of interest.

## References

[1] B. Liu , J. G. Zhang , W. Xu , Joule 2018, 2, 833.

[2] Y. Li , S. Guo , Matter 2021, 4, 1142.

[3] Z. Han , S. Li , Y. Wu , C. Yu , S. Cheng , J. Xie , J. Mater. Chem. A 2021, 9, 242150.

[4] A. Manthiram , Y. Fu , S. H. Chung , C. Zu , Y. S. Su , Chem. Rev. 2014, 114, 117517. 10.1021/cr500062v 25026475

[5] J. B. Goodenough , K. S. Park , J. Am. Chem. Soc. 2013, 135, 1167. 23294028 10.1021/ja3091438

[6] J. Guo , H. Pei , Y. Dou , S. Zhao , G. Shao , J. Liu , Adv. Funct. Mater. 2021, 31, 2010499.

[7] S. H. Chung , A. Manthiram , Joule 2018, 2, 710.

[8] R. Liu , S. Zhai , Z. Ye , M. Liu , Y. Xu , C. Li , X. Wang , T. Mei , J. Mater. Chem. A 2023, 11, 243307.

[9] Y. Wang , Y. Xiong , Q. Huang , Z. Bi , Z. Zhang , Z. Guo , X. Wang , T. Mei , J. Mater. Chem. A 2022, 10, 188666.

[10] Y. Hu , W. Chen , T. Lei , Y. Jiao , J. Huang , A. Hu , C. Gong , C. Yan , X. Wang , J. Xiong , Adv. Energy Mater. 2020, 10, 2000082.

[11] T. Wang , J. He , X. B. Cheng , J. Zhu , B. Lu , Y. Wu , ACS Energy Lett 2023, 8, 116.

[12] Y. Guo , H. Li , T. Zhai , Adv. Mater. 2017, 29, 1700007. 10.1002/adma.201700007 28585291

[13] G. Benveniste , H. Rallo , L. Canals Casals , A. Merino , B. Amante , J. Environ. Manage. 2018, 226, 1. 30103198 10.1016/j.jenvman.2018.08.008

[14] J. He , A. Manthiram , Energy Storage Mater 2019, 20, 55.

[15] S. Zhai , Z. Ye , R. Liu , H. Xu , C. Li , W. Liu , X. Wang , T. Mei , Adv. Funct. Mater. 2024, 34, 2314379.

[16] Z. Guan , X. Chen , F. Chu , R. Deng , S. Wang , J. Liu , F. Wu , Adv. Energy Mater. 2023, 13, 2302850.

[17] T. Tao , S. Lu , Y. Fan , W. Lei , S. Huang , Y. Chen , Adv. Mater. 2017, 29, 1700542. 10.1002/adma.201700542 28626966

[18] F. Chu , J. Liu , Z. Guan , R. Deng , L. Mei , F. Wu , Adv. Mater. 2023, 35, 2305470. 10.1002/adma.202305470 37566439

[19] Z. Ye , S. Zhai , R. Liu , M. Liu , Y. Xu , C. Li , X. Wang , T. Mei , Chem. Eng. J. 2024, 479, 147847.

[20] T. Liu , H. Hu , X. Ding , H. Yuan , C. Jin , J. Nai , Y. Liu , Y. Wang , Y. Wan , X. Tao , Energy Storage Mater 2020, 30, 346.

[21] H. Li , H. Yang , X. Ai , Adv. Mater. 2023, 35, 2305038. 10.1002/adma.202305038 37867204

[22] C. Li , R. Liu , Y. Xiao , F. Cao , H. Zhang , Energy Storage Mater 2021, 40, 439.

[23] Y. Zhao , Y. Ye , F. Wu , Y. Li , L. Li , R. Chen , Adv. Mater. 2019, 31, 1806532. 10.1002/adma.201806532 30672032

[24] X. Chen , T. Z. Hou , B. Li , C. Yan , L. Zhu , C. Guan , X. B. Cheng , H. J. Peng , J. Q. Huang , Q. Zhang , Energy Storage Mater 2017, 8, 194.

[25] Y. Liu , Y. Elias , J. Meng , D. Aurbach , R. Zou , D. Xia , Q. Pang , Joule 2021, 5, 2323.

[26] Z. X. Chen , M. Zhao , L. P. Hou , X. Q. Zhang , B. Q. Li , J. Q. Huang , Adv. Mater. 2022, 34, 2201555. 10.1002/adma.202201555 35475585

[27] F. Chu , M. Wang , J. Liu , Z. Guan , H. Yu , B. Liu , F. Wu , Adv. Funct. Mater. 2022, 32, 2205393.

[28] F. Wu , F. Chu , G. A. Ferrero , M. Sevilla , A. B. Fuertes , O. Borodin , Y. Yu , G. Yushin , Nano Lett 2020, 20, 5391. 32463248 10.1021/acs.nanolett.0c01778

[29] W. Chen , T. Qian , J. Xiong , N. Xu , X. Liu , J. Liu , J. Zhou , X. Shen , T. Yang , Y. Chen , C. Yan , Adv. Mater. 2017, 29, 1605160. 10.1002/adma.201605160 28165170

[30] Y. Gong , J. Li , K. Yang , S. Li , M. Xu , G. Zhang , Y. Shi , Q. Cai , H. Li , Y. Zhao , Nano‐Micro Lett. 2023, 15, 150. 10.1007/s40820-023-01120-7 PMC10247666 37286885

[31] S. Chen , Y. Gao , Z. Yu , M. L. Gordin , J. Song , D. Wang , Nano Energy 2017, 31, 418.

[32] M. Hagen , P. Fanz , J. Tübke , J. Power Sources 2014, 264, 30.

[33] Y. Ren , A. Bhargav , W. Shin , H. Sul , A. Manthiram , Angew. Chem. Int. Ed. 2022, 61, 202207907. 10.1002/anie.202207907 35796688

[34] X. Yu , J. Deng , R. Lv , Z. H. Huang , B. Li , F. Kang , Energy Storage Mater 2019, 20, 14.

[35] Z. Zhao , X. Duan , L. Zhang , Z. Che , K. Wang , B. Zheng , X. Wang , RSC Adv. 2021, 11, 307552. 10.1039/d1ra04281e PMC9041292 35498953

[36] Q. Lu , X. Zou , R. Ran , W. Zhou , K. Liao , Z. Shao , J. Mater. Chem. A 2019, 7, 224634.

[37] Y. Yao , H. Wang , H. Yang , S. Zeng , R. Xu , F. Liu , P. Shi , Y. Feng , K. Wang , W. Yang , et al., Adv. Mater. 2020, 32, 1905658. 10.1002/adma.201905658 31830338

[38] J. Xu , L. Xu , Z. Zhang , B. Sun , Y. Jin , Q. Jin , H. Liu , G. Wang , Energy Storage Mater 2022, 47, 223.

[39] Y. Ding , X. Li , Y. Chen , Y. Pi , J. Yu , L. Yuan , F. Wang , Chem. Eng. J. 2024, 482, 148803.

[40] D. Kong , W. Lv , R. Liu , Y. B. He , D. Wu , F. Li , R. Fu , Q. H. Yang , F. Kang , Energy Mater. Devices 2023, 1, 9370017.

[41] C. ZHANG , Q. Wang , Y. Song , G. Wang , H. Wang , Carbon 2023, 201, 76.

[42] Z. Zeng , W. Li , X. Chen , X. Liu , Adv. Funct. Mater. 2020, 30, 2004650.

[43] Z. Zhang , L. L. Kong , S. Liu , G. R. Li , X. P. Gao , Adv. Energy Mater. 2017, 7, 1602543.

[44] Q. Yu , Y. Lu , R. Luo , X. Liu , K. Huo , J. K. Kim , J. He , Y. In Luo , Adv. Funct. Mater. 2018, 28, 1804520.

[45] Z. Zhao , J. Wang , M. Cheng , J. Wu , Q. Zhang , X. Liu , C. Wang , J. Wang , K. Li , J. Wang , Electrochim. Acta 2020, 349, 136231.

[46] W. Wang , W. Dong , X. Hong , Y. Liu , S. Yang , Mater. Chem. Phys. 2022, 283, 126014.

[47] D. Qin , L. Wang , X. Zeng , J. Shen , F. Huang , G. Xu , M. Zhu , Z. Dai , Energy Storage Mater 2023, 54, 498.

[48] C. Jin , O. Sheng , W. Zhang , J. Luo , H. Yuan , T. Yang , H. Huang , Y. Gan , Y. Xia , C. Liang , J. Zhang , X. Tao , Energy Storage Mater 2018, 15, 218.

[49] Y. Wei , Y. Wang , X. Zhang , B. Wang , Q. Wang , N. Wu , Y. Zhang , H. Wu , ACS Appl. Mater. Interfaces 2020, 12, 350580. 10.1021/acsami.0c10047 32662619

[50] W. Zhang , B. Xu , L. Zhang , W. Li , S. Li , J. Zhang , G. Jiang , Z. Cui , H. Song , N. Grundish , et al., Small 2022, 18, 2105664. 10.1002/smll.202105664 34854562

[51] J. He , A. Manthiram , Adv. Energy Mater. 2020, 10, 2002654. 10.1002/aenm.202001972 PMC8216142 34158810

[52] S. Feng , Z. H. Fu , X. Chen , Q. Zhang , InfoMat 2022, 4, e12304.

[53] X. Chen , X. R. Chen , T. Z. Hou , B. Q. Li , X. B. Cheng , R. Zhang , Q. Zhang , Sci. Adv. 5, eaau7728.30793031

[54] J. He , A. L‐L Manthiram , Adv. Energy Mater. 2020, 10, 1903241. 10.1002/aenm.202001972 PMC8216142 34158810

[55] L. Liu , A. Corma , Chem. Rev. 2018, 118, 4981. 29658707 10.1021/acs.chemrev.7b00776 PMC6061779

[56] F. Feng , S. Han , Q. Lu , Q. Yun , Energy Mater. Devices 2023, 1, 9370008.

[57] F. Yao , J. Meng , X. Wang , J. Wang , L. Chang , G. Huang , Energy Mater. Devices 2023, 1, 9370020.

[58] Z. Ye , Y. Jiang , L. Li , F. Wu , R. Chen , Adv. Mater. 2021, 33, 2101204. 10.1002/adma.202101204 34245063

[59] D. K. Kim , J. B. Park , C. Choi , D. W. Kim , Chem. Eng. J. 2024, 479, 147820.

[60] K. Fan , H. Huang , Energy Storage Mater 2022, 50, 696.

[61] S. Liu , J. Li , X. Yan , Q. Su , Y. Lu , J. Qiu , Z. Wang , X. Lin , J. Huang , R. Liu , et al., Adv. Mater. 2018, 30, 1706895. 10.1002/adma.201706895 29423940

[62] J. Liu , R. Li , B. Yang , ACS Cent. Sci. 2020, 6, 2179. 33376780 10.1021/acscentsci.0c01306 PMC7760469

[63] F. Ma , K. Srinivas , X. Zhang , Z. Zhang , Y. Wu , D. Liu , W. Zhang , Q. Wu , Y. Chen , Adv. Funct. Mater. 2022, 32, 2206113.

[64] Z. Liang , J. Shen , X. Xu , F. Li , J. Liu , B. Yuan , Y. Yu , M. Zhu , Adv. Mater. 2022, 34, 2200102. 10.1002/adma.202200102 35238103

[65] D. Wang , K. Ma , J. Hao , W. Zhang , H. Shi , C. Wang , Z. Xiong , Z. Bai , Fu‐R Chen , J. Guo , B. Xu , X. Yan , Y. Gu , Chem. Eng. J. 2023, 466, 143182.

[66] Q. Yang , J. Cai , G. Li , R. Gao , Z. Han , J. Han , D. Liu , L. Song , Z. Shi , D. Wang , et al., Nat. Commun. 2024, 15, 3231. 38622167 10.1038/s41467-024-47565-1 PMC11018799

[67] X. Zhang , L. Zhou , K. Hu , D. Gao , S. Tang , L. He , Y. Chen , P. Zhang , Z. Zhang , Chem. Eng. J. 2023, 476, 146612.

[68] Y. Mo , X. Deng , P. Liu , J. Guo , W. Wang , G. Li , Mater. Sci. Eng. R. Rep. 2023, 152, 100711.

[69] J. Chang , J. Shang , Y. Sun , L. K. Ono , D. Wang , Z. Ma , Q. Huang , D. Chen , G. Liu , Y. Cui , et al., Nat. Commun. 2018, 9, 4480. 30367063 10.1038/s41467-018-06879-7 PMC6203774

[70] Q. Zhao , Q. Zhu , Y. Liu , B. Xu , Adv. Funct. Mater. 2021, 31, 2100457.

[71] L. Chen , L. Yue , X. Wang , S. Wu , W. Wang , D. Lu , X. Liu , W. Zhou , Y. Li , Small 2023, 19, 2206462. 10.1002/smll.202206462 36642788

[72] Y. Ren , B. Wang , H. Liu , H. Wu , H. Bian , Y. Ma , H. Lu , S. Tang , X. Meng , Chem. Eng. J. 2022, 450, 138046.

[73] R. Meng , Q. Du , N. Zhong , X. Zhou , S. Liu , S. Yin , X. Liang , Adv. Energy Mater. 2021, 11, 2102819.

[74] B. Dang , D. Gao , Y. Luo , Z. Zhang , J. Li , F. Wu , J. Energy Storage 2022, 52, 104981.

[75] S. Lv , X. Ma , S. Ke , Y. Wang , T. Ma , S. Yuan , Z. Jin , J. L. Zuo , J. Am. Chem. Soc. 2024, 146, 9385. 38512124 10.1021/jacs.4c01620

[76] Y. Li , S. Lin , D. Wang , T. Gao , J. Song , P. Zhou , Z. Xu , Z. Yang , N. Xiao , S. Guo , Adv. Mater. 2020, 32, 1906722. 10.1002/adma.201906722 31957092

[77] H. Shi , J. Qin , P. Lu , C. Dong , J. He , X. Chou , P. Das , J. Wang , L. Zhang , Z. S. Wu , Adv. Funct. Mater. 2021, 31, 2102314.

[78] M. Agostini , J. Y. Hwang , H. M. Kim , P. Bruni , S. Brutti , F. Croce , A. Matic , Y. K. Sun , Adv. Energy Mater. 8, 2018, 1801560.

[79] Y. Ouyang , W. Zong , X. Zhu , L. Mo , G. Chao , W. Fan , F. Lai , Y. E. Miao , T. Liu , Y. Yu , Adv. Sci. 2022, 9, 2203181. 10.1002/advs.202203181 PMC9475505 35863908

[80] X. Zhang , T. Yang , Y. Zhang , X. Wang , J. Wang , Y. Li , A. Yu , X. Wang , Z. Chen , Adv. Mater. 2023, 35, 2208470. 10.1002/adma.202208470 36469454

[81] J. Tan , J. Matz , P. Dong , J. Shen , M. Ye , Adv. Energy Mater. 2021, 11, 2100046.

[82] Y. Xiao , B. Han , Y. Zeng , S. S. Chi , X. Zeng , Z. Zheng , K. Xu , Y. Deng , Adv. Energy Mater. 2020, 10, 1903937.

[83] X. Chen , H. Ji , Z. Rao , L. Yuan , Y. Shen , H. Xu , Z. Li , Y. Huang , Adv. Energy Mater. 2022, 12, 2102774.

[84] A. Hu , W. Chen , X. Du , Y. Hu , T. Lei , H. Wang , L. Xue , Y. Li , H. Sun , Y. Yan , et al., Energy Environ. Sci. 2021, 14, 4115.

[85] M. Zhao , X. Chen , X. Y. Li , B. Q. Li , J. Q. Huang , Adv. Mater. 2021, 33, 2007298. 10.1002/adma.202007298 33586230

[86] Y. Gao , J. Gao , Z. Zhang , Y. Wu , X. Sun , F. Zhao , Y. Zhang , D. Song , W. Si , Q. Zhao , et al., ACS Appl. Mater. Interfaces 2024, 16, 188434. 10.1021/acsami.4c00358 38586920

[87] X. Zhang , H. Zhang , Y. Geng , Z. Shi , S. Zhu , Q. Xu , Y. Min , Chem. Eng. J. 2022, 444, 136328.

[88] Y. Lin , X. Wang , J. Liu , J. D. Miller , Nano Energy 2017, 31, 478.

[89] X. Yin , L. Wang , Y. Kim , N. Ding , J. Kong , D. Safanama , Y. Zheng , J. Xu , D. V. M. Repaka , K. Hippalgaonkar , S. W. Lee , S. Adams , G. W. Zheng , Adv. Sci. 2020, 7, 2001303. 10.1002/advs.202001303 PMC7539184 33042749

[90] S. Chen , B. Ding , Q. Lin , Y. Shi , B. Hu , Z. Li , H. Dou , X. Zhang , J. Electroanal. Chem. 2021, 880, 114874.

[91] S. Lian , Y. Wang , H. Ji , X. Zhang , J. Shi , Y. Feng , X. Qu , Nanomaterials 2021, 11, 2562. 34685002 10.3390/nano11102562 PMC8540722

[92] Z. Zhang , B. Zhao , S. Zhang , J. Zhang , P. Han , X. Wang , F. Ma , D. Sun , Y. Jin , K. Kanamura , G. Cui , J. Power Sources 2021, 487, 229428.

[93] L. Wang , X. Yin , C. Jin , C. Lai , G. Qu , G. W. Zheng , ACS Appl. Energy Mater. 2020, 3, 115407.

[94] X. Tao , Y. Liu , W. Liu , G. Zhou , J. Zhao , D. Lin , C. Zu , O. Sheng , W. Zhang , H. W. Lee , et al., Nano Lett 2017, 17, 2967. 28388080 10.1021/acs.nanolett.7b00221

[95] X. Xu , G. Hou , X. Nie , Q. Ai , Y. Liu , J. Feng , L. Zhang , P. Si , S. Guo , L. Ci , J. Power Sources 2018, 400, 212.

[96] Y. X. Song , Y. Shi , J. Wan , S. Y. Lang , X. C. Hu , H. J. Yan , B. Liu , Y. G. Guo , R. Wen , L. J. Wan , Energy Environ. Sci. 2019, 12, 2496.

[97] X. Zhu , Z. Wen , Z. Gu , Z. Lin , J. Power Sources 2005, 139, 269.

[98] L. Chen , L. Z. Fan , Energy Storage Mater 2018, 15, 37.

[99] X. Li , D. Wang , H. Wang , H. Yan , Z. Gong , Y. Yang , ACS Appl. Mater. Interfaces 2019, 11, 227453. 10.1021/acsami.9b05212 31190524

[100] Y. Zhang , Y. Zhao , D. Gosselink , P. Chen , Ionics 2015, 21, 381.

[101] R. Fang , H. Xu , B. Xu , X. Li , Y. Li , J. B. Goodenough , Adv. Funct. Mater. 2021, 31, 2001812.

[102] Z. Zhang , Y. Dong , Y. Gu , P. Lu , F. Xue , Y. Fan , Z. Zhu , J. Lin , Q. Li , Z. S. Wu , J. Mater. Chem. A 2022, 10, 9515.

[103] M. A. Zaed , K. H. Tan , N. Abdullah , R. Saidur , A. K. Pandey , A. M. Saleque , Open Ceram 2024, 17, 100526.

[104] X. Zhu , L. Lin , M. Pang , C. Jia , L. Xia , G. Shi , S. Zhang , Y. Lu , L. Sun , F. Yu , et al., Nat. Commun. 2024, 15, 3218. 38622151 10.1038/s41467-024-47603-y PMC11018853

